# Linking NRP2 With EMT and Chemoradioresistance in Bladder Cancer

**DOI:** 10.3389/fonc.2019.01461

**Published:** 2020-01-21

**Authors:** Alexander Schulz, Ielizaveta Gorodetska, Rayk Behrendt, Susanne Fuessel, Kati Erdmann, Sarah Foerster, Kaustubh Datta, Thomas Mayr, Anna Dubrovska, Michael H. Muders

**Affiliations:** ^1^Faculty of Medicine and University Hospital Carl Gustav Carus, OncoRay-National Center for Radiation Research in Oncology, Technische Universität Dresden and Helmholtz-Zentrum Dresden-Rossendorf, Dresden, Germany; ^2^Faculty of Medicine, Institute for Immunology, Technische Universität Dresden, Dresden, Germany; ^3^Department of Urology, Technische Universität Dresden, Dresden, Germany; ^4^Rudolf Becker Laboratory for Prostate Cancer Research, Center of Pathology, University of Bonn Medical Center, Bonn, Germany; ^5^Department of Biochemistry and Molecular Biology, University of Nebraska Medical Center, Omaha, NE, United States; ^6^Helmholtz-Zentrum Dresden - Rossendorf, Institute of Radiooncology – OncoRay, Dresden, Germany; ^7^German Cancer Consortium (DKTK), Partner Site Dresden, Dresden, Germany; ^8^German Cancer Research Center (DKFZ), Heidelberg, Germany

**Keywords:** bladder cancer, Neuropilin-2 (NRP2), glioma-associated oncogene family zinc finger 2 (GLI2), secreted phosphoprotein 1 (SPP1), osteopontin (OPN), epithelial-to-mesenchymal transition (EMT), RT112, J82

## Abstract

Neuropilin-2 (NRP2) is a prognostic indicator for reduced survival in bladder cancer (BCa) patients. Together with its major ligand, vascular endothelial growth factor (VEGF)-C, NRP2 expression is a predictive factor for treatment outcome in response to radiochemotherapy in BCa patients who underwent transurethral resection. Therefore, we investigated the benefit of combining cisplatin-based chemotherapy with irradiation treatment in the BCa cell line RT112 exhibiting or lacking endogenous NRP2 expression in order to evaluate NRP2 as potential therapeutic target. We have identified a high correlation of NRP2 and the glioma-associated oncogene family zinc finger 2 (GLI2) transcripts in the cancer genome atlas (TCGA) cohort of BCa patients and a panel of 15 human BCa cell lines. Furthermore, we used *in vitro* BCa models to show the transforming growth factor-beta 1 (TGFβ1)-dependent regulation of NRP2 and GLI2 expression levels. Since NRP2 was shown to bind TGFβ1, associate with TGFβ receptors, and enhance TGFβ1 signaling, we evaluated downstream signaling pathways using an epithelial-to-mesenchymal transition (EMT)-assay in combination with a PCR profiling array containing 84 genes related to EMT. Subsequent target validation in NRP2 knockout and knockdown models revealed secreted phosphoprotein 1 (SPP1/OPN/Osteopontin) as a downstream target positively regulated by NRP2.

## Introduction

Bladder cancer (BCa) is the 9th most common malignancy in the world with the highest incidence in Europe and North America (1). There are three main stages of this disease, the non-muscle invasive bladder cancer (NMIBC), the muscle invasive bladder cancer (MIBC), and the metastatic BCa. At diagnosis, 70% of the patients present with NMIBC, 20% with MIBC, and 10% with metastatic disease (2). While NMIBC can be treated with good outcome by transurothelial resection of the bladder tumor (TURBT) and adjuvant intravesical Bacillus Calmette-Guérin (BCG) or chemotherapy, the treatment options for the more aggressive MIBC consist of neoadjuvant and adjuvant cisplatin treatment and radical cystectomy. Despite the aggressive therapy regimen, MIBC has a 50% risk to progress to metastatic disease. The average survival time in these patients is 14–15 months (3), and no curative treatment option is available for these patients. Only recently, immune checkpoint inhibition has become available in patients with metastatic disease. The success rate for this treatment is still uncertain. Nevertheless, new therapy options are urgently needed for this disease stage. Radiochemotherapy has emerged as a promising new option for improving locoregional control and being able to preserve the bladder and hence quality of life (4–6).

Our group previously demonstrated that Neuropilin-2 (NRP2) is a prognostic indicator for reduced survival in BCa patients. Together with its major ligand, vascular endothelial growth factor (VEGF)-C, NRP2 expression is capable of predicting treatment outcome in response to radiochemotherapy in BCa patients who underwent transurethral resection (7). NRP2 is a co-receptor frequently overexpressed in cancers. Because NRP2 expression is significantly associated with poor prognosis in renal cell carcinomas, colorectal carcinomas, gastric carcinomas, osteosarcoma, breast, pancreatic, and bladder cancer (7–13), it has become an attractive target for cancer therapy. This is in part due to the fact that NRP2 is implicated in signaling pathways commonly hijacked by tumor cells.

Hedgehog (Hh) signaling is silenced in many adult tissues; however, during tumorigenesis, it is often reactivated (14). Canonical Hh signaling is induced by sonic, indian, or desert Hh ligands and functions via glioma-associated oncogene family zinc finger (GLI) proteins, the major transcriptional effectors of Hh signaling (14). GLI proteins contain activation (GLI1, GLI2, and GLI3) and repression domains (GLI2 and GLI3) (15), thus differentially affecting their downstream target genes. Hh signaling can also be induced by non-canonical pathways including transforming growth factor (TGF)β-induced signaling (14, 16, 17). Non-canonical Hh signaling by TGFβ (and Wnt) was shown to induce GLI2 expression and activation (14). In another non-canonical pathway, NRP2 also directly enhances Hh signaling in a ligand-independent manner (18).

Epithelial-to-mesenchymal transition (EMT), a complex molecular process, plays an important role in tumor progression, invasion, and metastasis and is induced, among others, by TGFβ (19). EMT signaling is associated with therapy resistance in various tumor entities, including breast cancer (20), pancreatic cancer (21), and BCa (22). Interestingly, TGFβ1-induced EMT highly increased NRP2 protein levels and NRP2 was subsequently identified as a receptor for both the latent and active form of TGFβ1 (23).

Taken together, NRP2 supports a vast number of tumor-promoting events but seems to be less crucial in most healthy tissues, and thus, it has become an attractive target for anti-cancer therapy. In this report, we aimed to elucidate NRP2's role in TGFβ-mediated EMT as well as in radio(chemo)therapy treatment of BCa models.

## Results

### The Relationship of NRP2 and GLI2 in BCa

**Figure 1:**
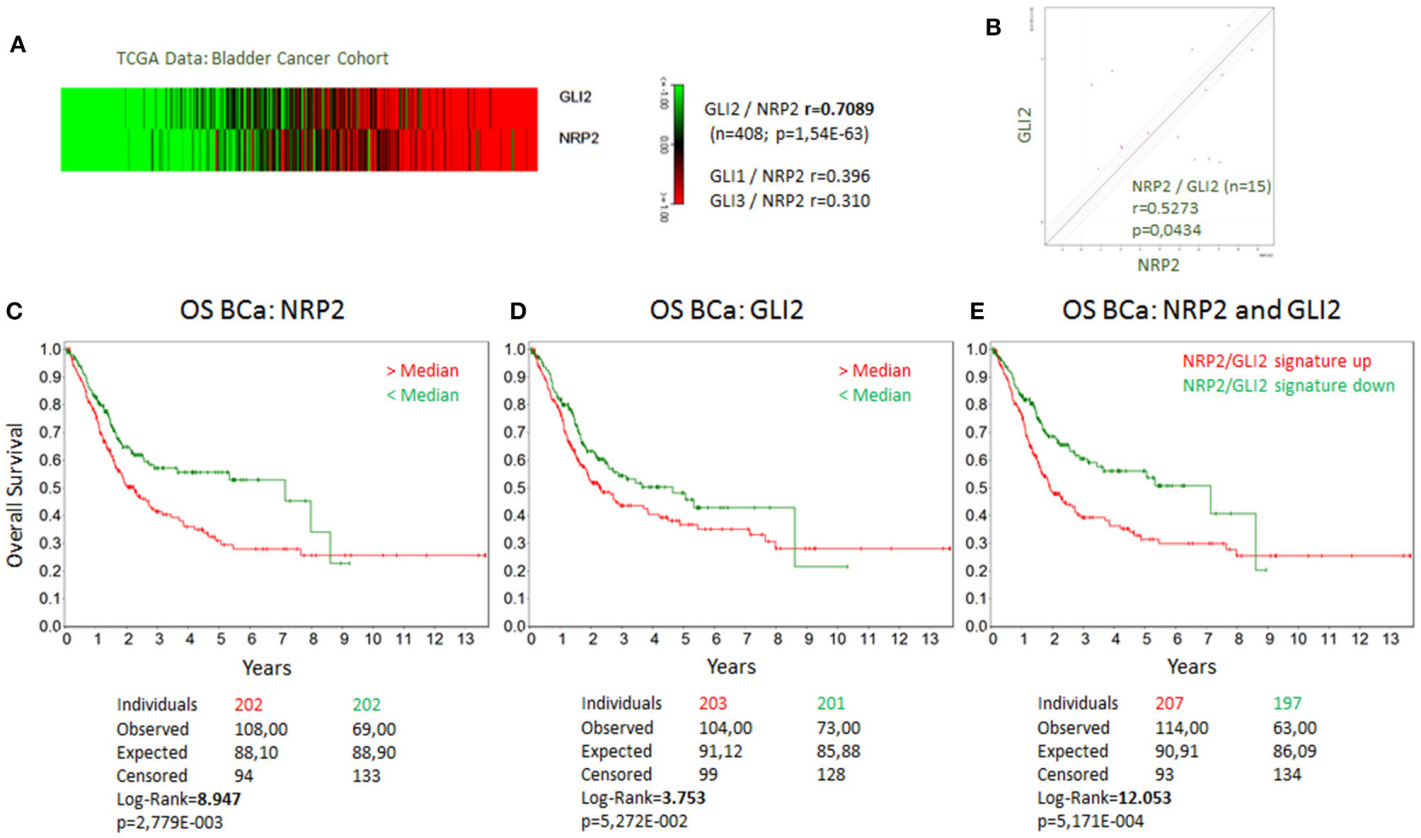
Coexpression of GLI2 and NRP2 genes in BCa cells and correlation of GLI2 and NRP2 gene expression with overall survival of BCa patients. **(A)** Correlation of GLI2 and NRP2 gene expression in a provisional bladder cancer cohort of The Cancer Genome Atlas (TCGA). Correlation coefficient of GLI1 and GLI3 to NRP2 from the same data set is provided for comparison. **(B)** mRNA expression of NRP2 and GLI2 was correlated in a panel of 15 bladder cancer cell lines. Normalized to housekeeping gene HPRT1. Kaplan-Meier plot of overall survival (OS) of bladder cancer (BCa) patients with high (red) compared to low mRNA signature (green) for NRP2 **(C)**, GLI2 **(D)**, and combined NRP2/GLI2 **(E)**. Log-Rank value was increased compared to single gene signature. The same applies for disease-free survival shown in Supplementary Figure 2.

**Figure 2:**
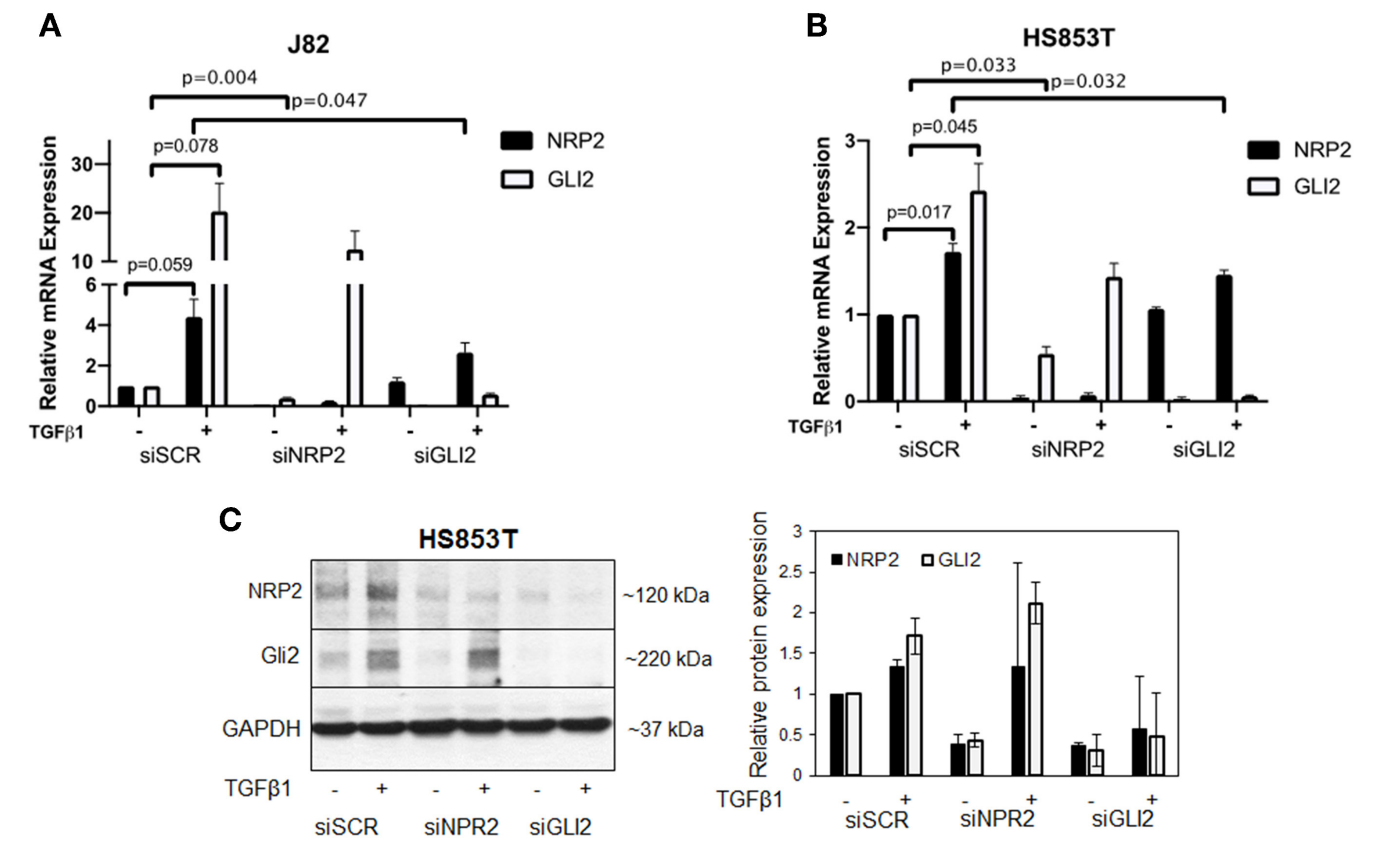
Validation of the relationship of NRP2 and GLI2 in BCa cells after NRP2 and GLI2 knockdown. Quantitative real-time PCR in two cell lines: J82 **(A)** and HS853T **(B)** with robust expression of NRP2 and GLI2 were subjected to knockdown of these gene products (siNRP2 or siGLI2) or scrambled control (siSCR) and treated with 5 ng/ml TGFβ1 or left untreated (±). All transcripts were induced by TGFβ1 treatment. GLI2 levels were reduced after NRP2 knockdown while NRP2 induction by TGFβ1 is inhibited following GLI2 knockdown. Normalized to housekeeping gene HPRT1 and plotted relative to untreated siSCR sample. Significance calculated by two-way ANOVA. Error bars indicate standard error of the mean. *n* = 3. Not all *p* values are shown. A plot of all targets including NRP2a, NRP2b, and GLI1 is provided in Supplementary Figure 3. The figure also contains additional graphs with normalization against two other housekeeping genes ACTB and GAPDH. All *p* values for all cell lines are provided in Supplementary Table 2. **(C)** Western blot analysis of NRP2 and GLI2 expression in HS853T cells in response to GLI2 or NRP2 knockdown. Cells were transfected with gene-specific siRNA (siNRP2 or siGLI2) or siSCR and treated with 5 ng/ml TGFβ1 or left untreated (±). Relative protein expression was normalized to GAPDH. Error bars indicate standard deviation. *n* = 2.

Because NRP2 has previously shown to enhance TGFβ signaling, we first aimed to determine the correlation of NRP2 mRNA expression with the expression of other TGFβ regulated genes in bladder tumors. To achieve this aim, we employed data from 408 BCa patients of the provisional BCa cohort from The Cancer Genome Atlas (TCGA) data set. The complete list of genes can be found in Supplementary Tables 1A,B. One of the most interesting identified targets was the Hh transcription factor GLI2 (*r* = 0.709). It was more strongly associated with NRP2 expression than its related genes GLI1 (*r* = 0.396) or GLI3 (*r* = 0.310) (Figure 1A). Notably, this relation was confirmed in other TCGA data sets of breast and prostate cancer (Supplementary Figures 1A,B). Furthermore, we confirmed this strong correlation between NRP2 and GLI2 transcripts by qPCR in a panel of 15 human BCa cell lines (Figure 1B and Supplementary Figure 1C) and by analysis of NRP2 and GLI2 co-expression in the cell lines of urinary tract (*n* = 26) using RNA-sequencing (RNA-seq) data from the Broad Institute Cell Line Encyclopedia (Supplementary Figure 1D). In order to investigate the potential clinical impact of NRP2 and GLI2 expression levels, we compared overall survival of single gene signatures of either NRP2 or GLI2 to the combined NRP2/GLI2 signature in Kaplan–Meier plots with median separation. This analysis demonstrated that combining NRP2 and GLI2 gene expression results in a higher predictive value for overall survival (Figures 1C–E). Notably, the same trend was observed for disease-free survival (Supplementary Figure 2). This observation, together with the strong correlation of both transcripts, tempted us to investigate the relationship of NRP2 and GLI2 in more detail by selecting two BCa cell lines, namely, J82 and HS853T, showing robust mRNA levels of both NRP2 and GLI2 (Supplementary Figure 1C) for knockdown experiments. To further evaluate the role of NRP2 in TGFβ-induced EMT, we treated these cell lines with TGFβ1 in addition to the respective knockdown. siRNA-mediated knockdown of NRP2 resulted in a reduction of GLI2 expression in both cell lines. On the other hand, induction of NRP2 expression by TGFβ1 is impaired following GLI2 knockdown (Figure 2). This suggests a co-dependency of both targets based on the ligand initiating the downstream pathways. Notably, we also checked the expression of isoforms NRP2a and NRP2b as well as GLI1, a direct target gene of GLI2 (Supplementary Figures 3, 4). As expected, GLI1 expression was also induced in response to TGFβ1 but to a lesser extent than GLI2. Accordingly, GLI1 levels were reduced following GLI2 knockdown. Isoforms NRP2a and NRP2b were induced similarly in TGFβ1-treated samples and GLI2 knockdown led to a shift of these isoforms in favor of NRP2b. A complete list of all *p* values for all targets and samples is provided in Supplementary Table 2.

**Figure 3:**
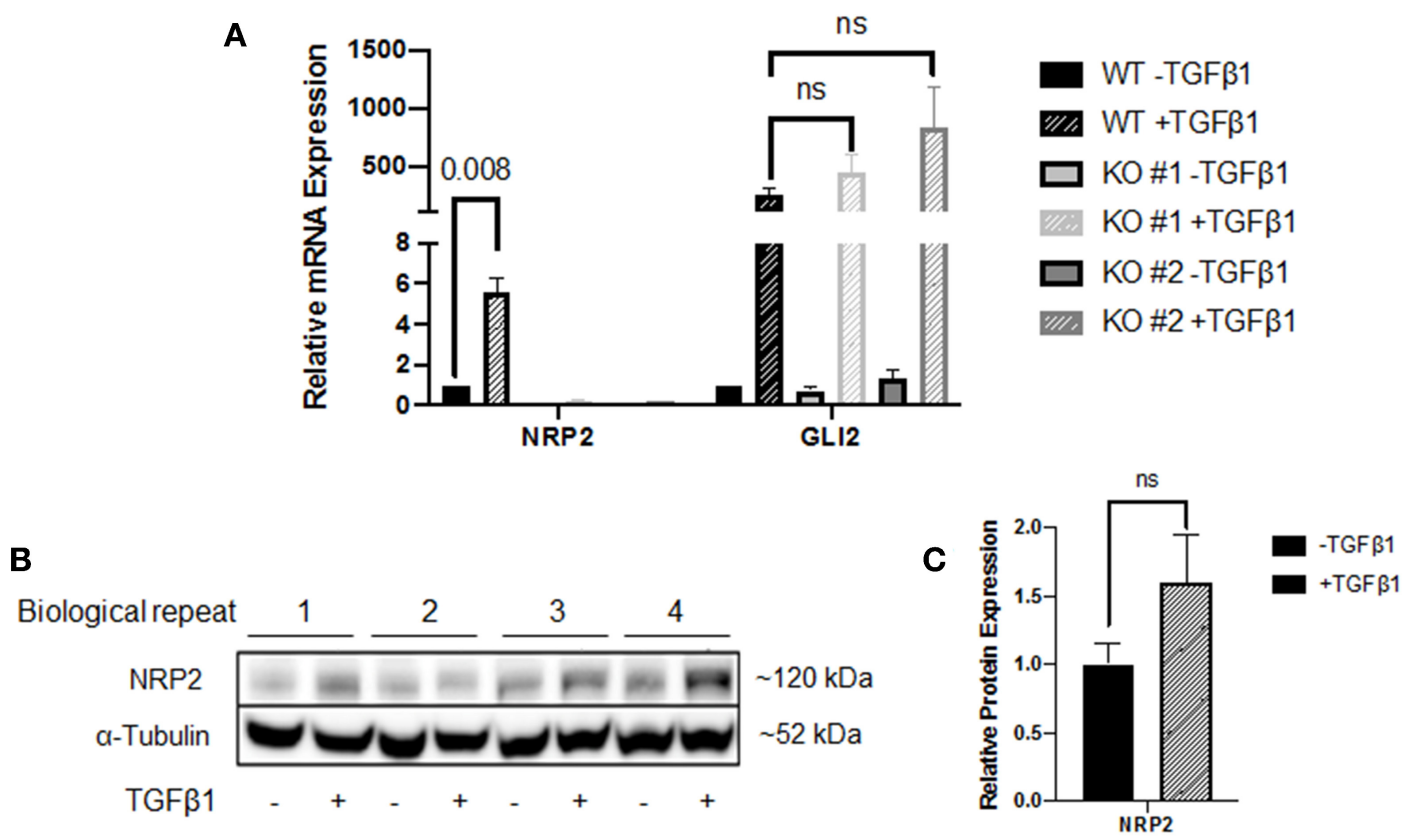
Analysis of the NRP2 and GLI2 relationship in BCa cells after NRP2 knockout. **(A)** mRNA expression of NRP2 and GLI2 transcript in response to TGFβ1 treatment of two independent NRP2 knockout clones (KO #1 and KO #2) and their parental wild-type BCa cell line RT112 (WT). Untreated samples were used as control. NRP2 transcripts are highly induced by TGFβ1 only when NRP2 protein is expressed. Normalized to housekeeping gene HPRT1. Significance calculated by two-way ANOVA. Error bars indicate standard error of the mean. *n* = 4. **(B)** Western blot of WT cell line for NRP2 and α-tubulin as loading control. **(C)** Calculation of optical densitometry. Significance determined by two-tailed, unpaired Student's *t* test. *n* = 4.

In addition to the NPR2 knockdown in these cell lines, we created two NRP2 knockout clones from the cell line RT112. Wild-type (WT) and knockout (KO) cells were subjected to treatment with TGFβ1 and compared to untreated controls. KO cell lines may still produce NRP2 transcript but the resulting mRNA contains premature translational stop codons on all alleles. While WT cells significantly increased the level of NRP2 mRNA in response to TGFβ1, both KO clones failed to upregulate transcription, potentially hinting to a positive feedback loop of NRP2 enhancing its own transcription upon TGFβ-signaling (Figure 3A). Moreover, TGFβ1 highly induced GLI2 in both WT and KO cell lines, suggesting that NRP2 is not upstream of TGFβ1-mediated GLI2 regulation. The mRNA level of GLI2 in WT cells was comparable to both KOs in the untreated state. In the treated samples, TGFβ1 induced GLI2 transcription more prominently in KO cell lines. However, this difference was not significant. Hence, TGFβ1-induced GLI2 expression seems to be independent of NRP2 in this model or cell line.

The fact that NRP2 is induced by more than 5-fold in WT cells raised the question whether upregulation is a direct effect of TGFβ signaling or TGFβ1 leads to faster degradation of NRP2, which may prompt cells to upregulate its transcription for maintaining constant NRP2 protein levels. Therefore, we performed Western blot analysis of WT lysates, which indicated that NRP2 protein was not upregulated significantly in TGFβ1-treated samples compared to untreated samples (Figures 3B,C and Supplementary Figure 5A). Despite the minor increase on protein level, it is not comparable to the 5-fold upregulation of NRP2 transcript, suggesting that the effect on mRNA level may potentially be a compensatory mechanism.

### Knockout of NRP2 Alters Gene Expression of EMT Regulators

**Figure 4:**
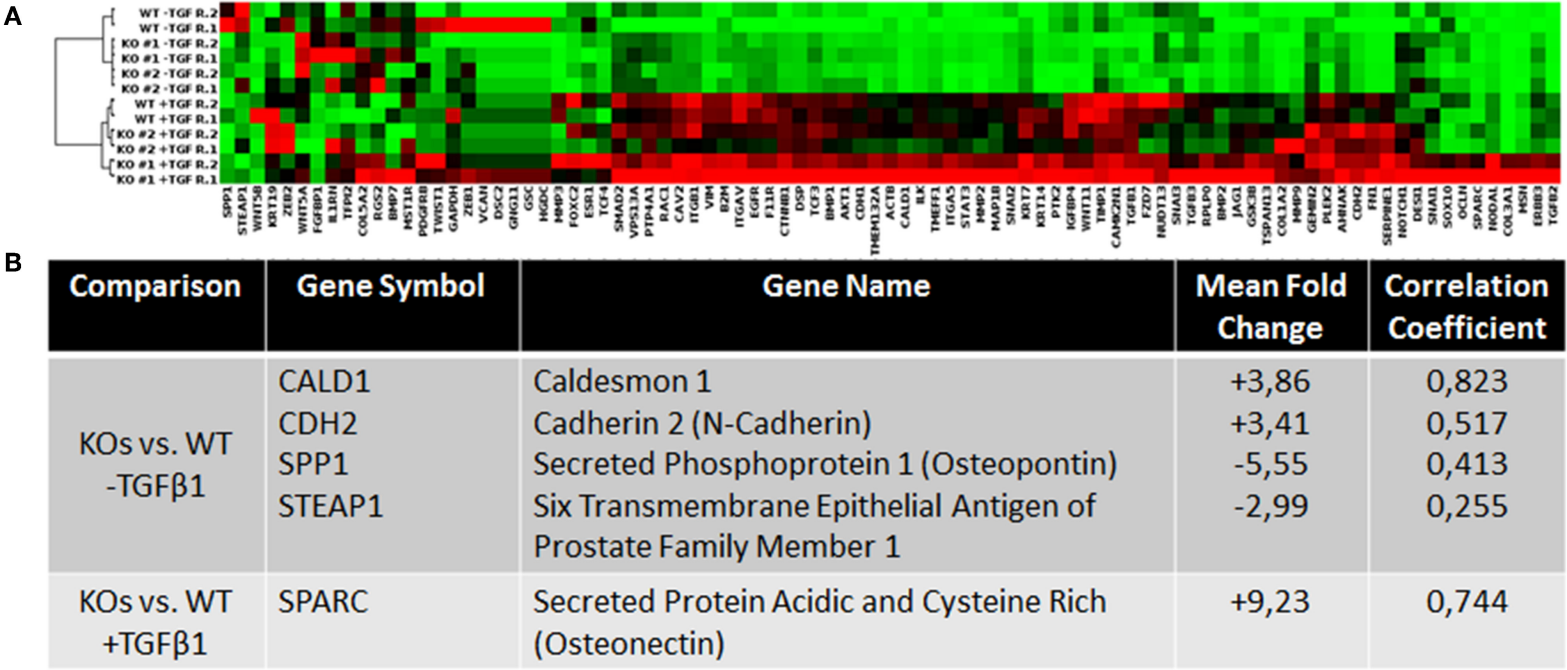
Analysis of the expression levels of EMT-related genes in wild type and NRP2 knockout BCa cells using PCR gene expression array. **(A)** Clustergram for all samples and genes on the PCR array plate extracted from Qiagen data analysis center software. **(B)** Consistently deregulated genes in both KOs vs. WT samples and their correlation based on the TCGA data set. *n* = 2.

**Figure 5:**
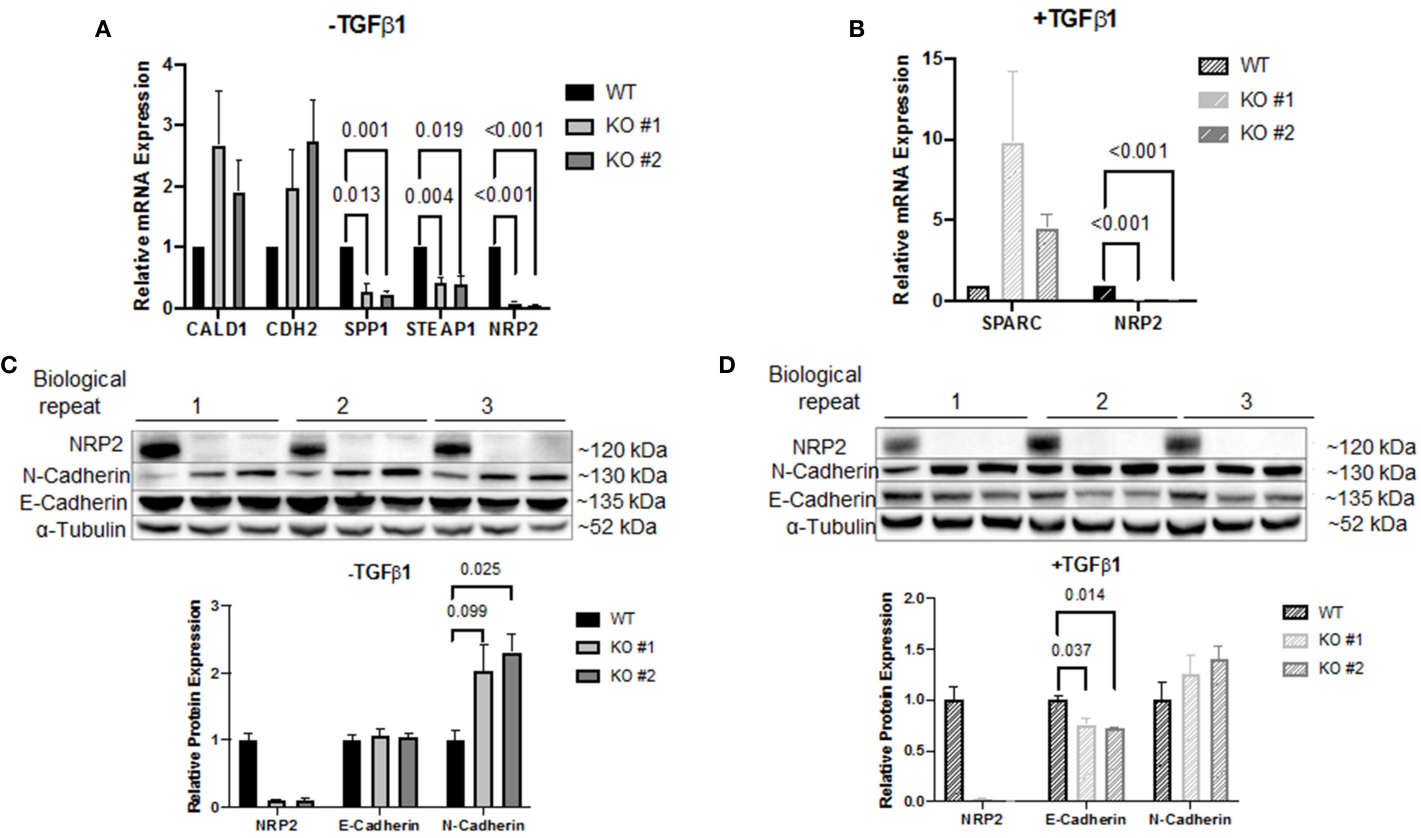
Validation of the PCR array results. Target validation by qPCR in two NRP2 knockout clones (KO #1, KO #2) compared to their parental wild-type cell line RT112 (WT) **(A)** without TGFβ1 treatment or **(B)** including TGFβ1 treatment. Genes SPP1 and STEAP1 were significantly affected by NRP2 knockout in both knockout clones. For other targets, data were either inconsistent or not significant. Data were normalized to housekeeping gene HPRT1. Significance calculated by two-way ANOVA. Error bars indicate standard error of the mean. *n* = 4. **(C)** Western blot of samples that remained untreated for proteins NRP2, N-Cadherin, and E-Cadherin. α-Tubulin served as loading control. Untreated KO cells demonstrated increased N-Cadherin expression. **(D)** Western blot of TGFβ1-treated KO clones did not show a significantly upregulated N-Cadherin expression compared to parental cells but revealed downregulated E-Cadherin on protein level. Significance calculated by two-way ANOVA. Error bars indicate standard error of the mean. *n* = 3.

To investigate how NRP2 may enhance TGFβ-signaling, cDNA of WT and KO cell lines was analyzed by a PCR array covering 84 genes involved in EMT. By addition of TGFβ1, EMT was successfully and similarly induced in both KO clones and their wild-type parental cell line RT112 as visible by an increase in the EMT marker vimentin (Supplementary Figure 5B). With this approach, it was possible to identify four genes whose expression was altered in both KOs compared to WT without TGFβ1 treatment. When all cell lines received TGFβ1 treatment, one gene was found to be deregulated in KOs vs. WT cells (Figure 4). Validation of these targets by qPCR in four biological repeats demonstrated that upregulation of Caldesmon 1 (CALD1) and Cadherin 2 (CDH2, N-Cadherin) was not significant but Secreted Phosphoprotein 1 (SPP1) and Six Transmembrane Epithelial Antigen of Prostate Family Member 1 (STEAP1) mRNA levels were significantly downregulated in KO clones (Figure 5A). When cells were treated with TGFβ1, mRNA expression of Secreted Protein Acidic and Cysteine Rich (SPARC) was highly upregulated but remained non-significant (Figure 5B). As an example, SPP1 expression as determined by the human EMT PCR array is lower in both KO clones compared to the wild-type parental cell line (Supplementary Figure 6).

CDH2 was the only target that demonstrated robust mRNA expression in both conditions. Therefore, validation on protein level seemed promising only for this target. Western blot analysis demonstrated that N-Cadherin (gene CDH2) appeared to be upregulated following knockout of NRP2 without TGFβ1 treatment (Figure 5C). Since E-Cadherin is known to be an opposing player of N-Cadherin in EMT, we used this target as control. Surprisingly, lysates from TGFβ1-treated samples showed significantly decreased levels of E-Cadherin in KOs compared to WT despite no change in gene expression was detected by the PCR array (Figure 5D). qPCR of CDH1 (E-Cadherin) confirmed that this change did not arise from altered transcript levels (Supplementary Figure 7). Because EMT-related signaling pathways are involved in the regulation of cancer stem cell (CSC) phenotype and properties in urothelial carcinoma including BCa, we analyzed if the absence of NRP2 has an impact on the CSC-related properties (24). Sphere forming assays revealed that the number of spheres formed by KO cells was significantly reduced (Supplementary Figure 8A).

**Figure 6:**
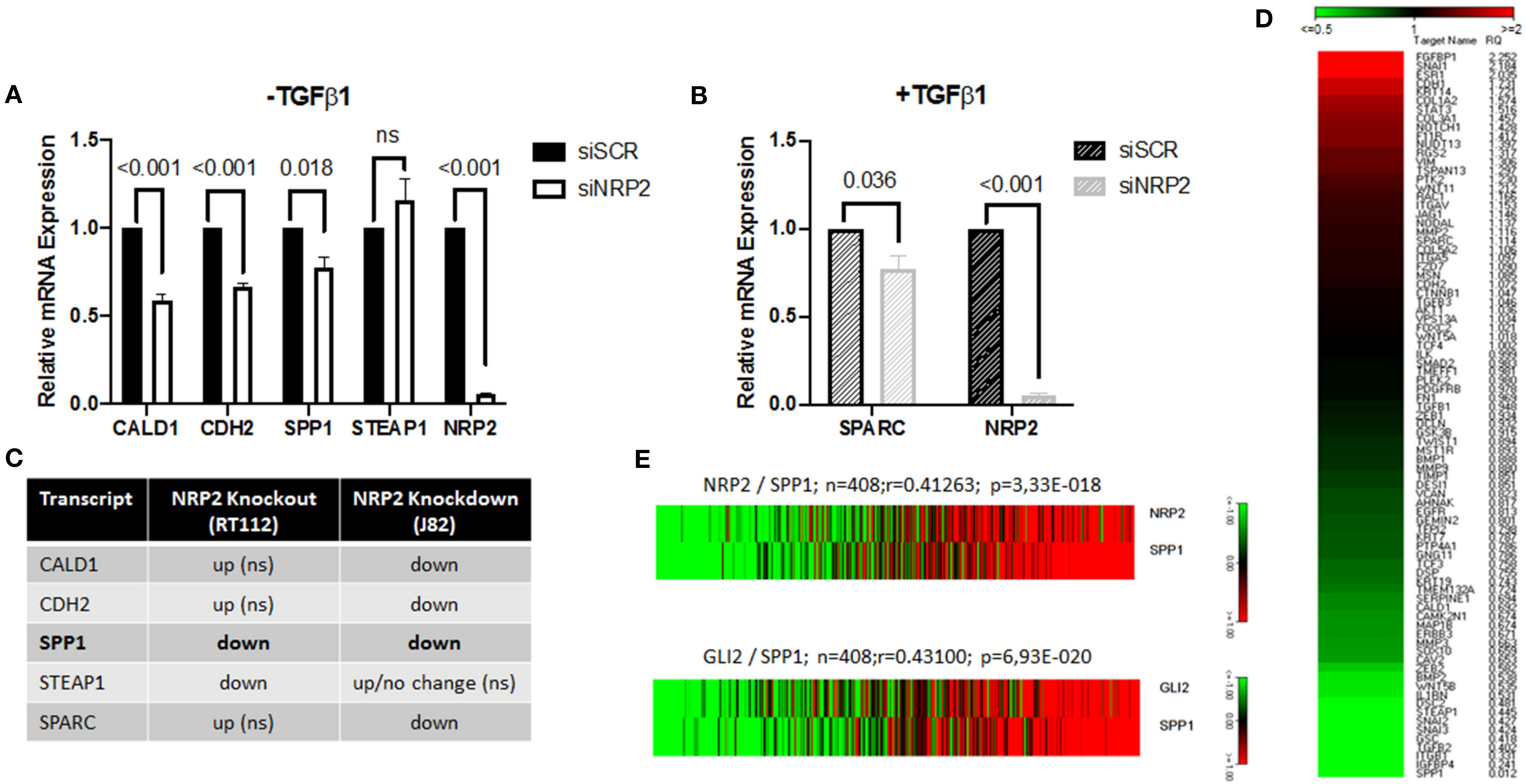
Identification of SPP1 as one of the NRP2-regulated and EMT-associated genes. qPCR of all identified targets following NRP2 knockdown in cell line J82 **(A)** excluding or **(B)** including TGFβ1 treatment. Significance calculated by two-way ANOVA. Error bars indicate standard error of the mean. *n* = 3. **(C)** Comparison of deregulated genes by NRP2 knockout in RT112 or NRP2 knockdown in J82. SPP1 is the only gene reacting the same way to both depletions in two different cell lines. ns = not significant. **(D)** Analysis of J82 cells transfected with siSCR (three pooled biological repeats) and J82 cells transfected with siSPP1 (three pooled biological repeats) with EMT PCR array. **(E)** Correlation of NRP2 with SPP1 and GLI2 with SPP1 in a provisional bladder cancer cohort of The Cancer Genome Atlas (TCGA).

To confirm the aberrant EMT signature in an additional cell line, we used conventional siRNA-mediated knockdown of NPR2 in the BCa cell line J82 and HS853T (Figures 6A,B and Supplementary Figure 8B). The results show that all except one gene (STEAP1) were significantly altered by NRP2 knockdown (Figures 6A,B). Analysis of BCa TCGA dataset also revealed that all validated genes positively correlate with expression of both NRP2 and GLI2 genes (Supplementary Figure 8C). However, only one gene transcript was regulated in a similar manner to NRP2 knockout in RT112 as well as NRP2 knockdown in J82 (Figure 6C). The SPP1 gene (Osteopontin, OPN) was previously reported to be induced by VEGF (25) and to be associated with decreased survival, disease stage, and grading in BCa (26, 27). Previous findings support the role of SPP1 as one of the key EMT regulators (28). We applied an EMT PCR array to analyze if SPP1 regulates gene expression of EMT regulators in our cell models. We found that SPP1 knockdown in J82 cells decreased expression of a number of key EMT genes including SNAI1, COL1A2, FGFBP1, and STAT3 (Figure 6D). In the TCGA BCa cohort, SPP1 expression positively correlated with both NRP2 and GLI2 (Figure 6E). Although our data did not confirm that SPP1 might be a regulator of BCa radiosensitivity on its own, analysis of TCGA BCa dataset showed that combined NRP2/SPP1 signature improved predictive value for disease-free but not overall survival compared to single NRP2 gene expression (Supplementary Figures 8D,E, 9, 10).

### The Relevance of NRP2 to Treatment With Radiochemotherapy

**Figure 7:**
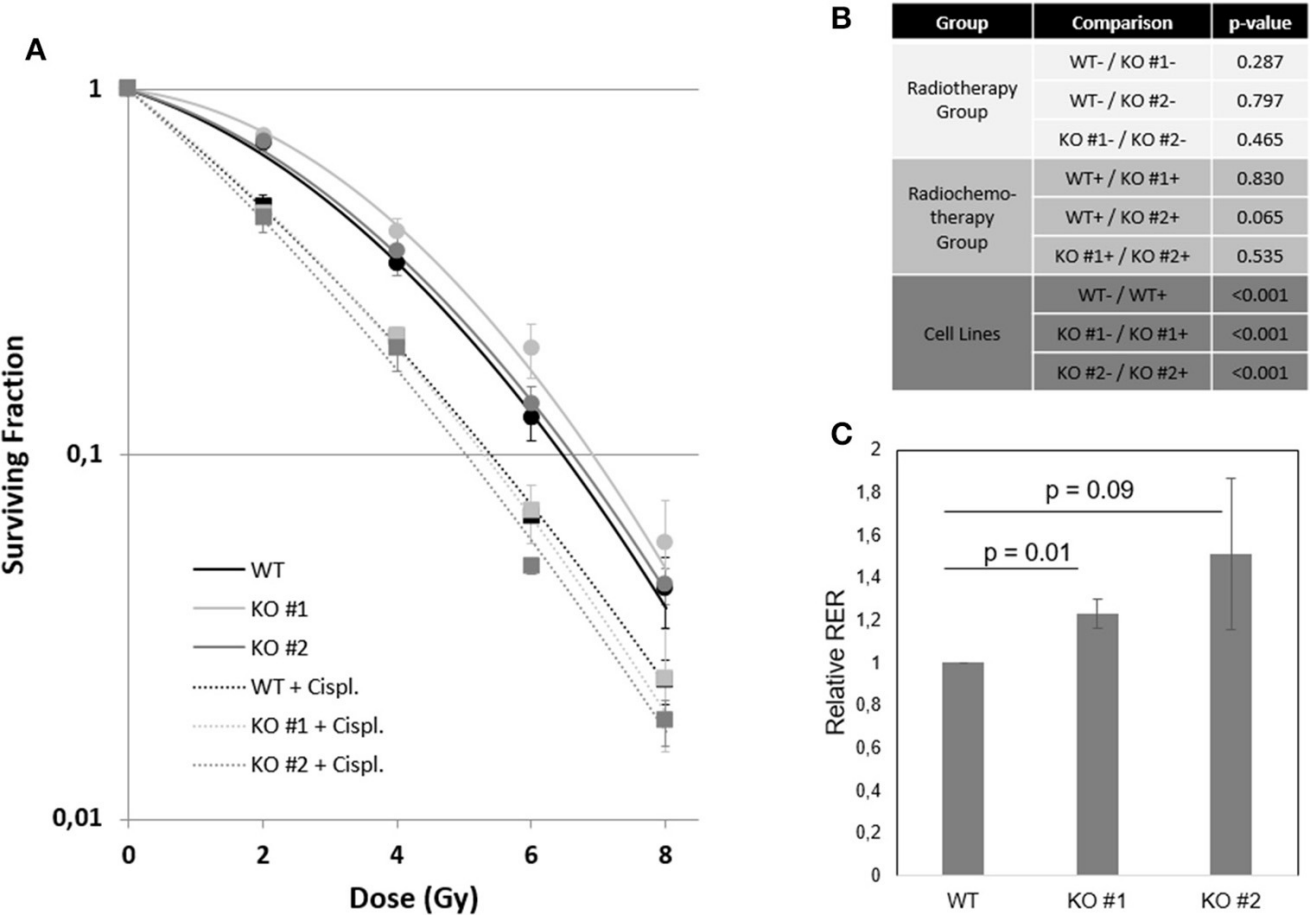
NRP2 regulates BCa radiochemosensitivity. **(A)** Surviving fraction of cell line RT112 either harboring (WT—black) or lacking (KO #1—light gray, KO #2—dark gray) endogenous NRP2 after radiotherapy treatment with doses of 0, 2, 4, 6, and 8Gy (dots). All cell lines were additionally treated with the chemotherapeutic drug cisplatin (Cispl. IC5 for KO #2 = 1.52 μM) for 24 h prior to radiation (squares). Plotted lines were fitted using the linear quadratic model for both the radiochemotherapy group (dashed lines) and the radiation only group (solid lines). Error bars indicate standard error of the mean. *n* = 3. **(B)** Table of significance of fitted curves. Significance was calculated by SPSS software. –, without cisplatin (Cispl.); +, with cisplatin. **(C)** Radiation enhancement ratios (RERs) were calculated at doses 2, 4, 6, and 8Gy from interpolation of linear-quadratic cell survival curves using mean values of three independent experiments. Relative RER values for KO #1 and KO #2 were calculated as *RERrel* = *Average* ([*RER* *KO*, 2*Gy*]/[*RER* *WT*, 2*Gy*];[*RER* *KO*, 4*Gy*]/[*RER* *WT*, 4*Gy*];[*RER* *KO*, 6*Gy*]/[*RER* *WT*, 6*Gy*];[*RER* *KO*, 8*Gy*]/[*RER* *WT*, 8*Gy*]). Error bars indicate standard error of the mean. *n* = 3.

The standard curative treatment of BCa is surgery and chemotherapy. Only for progressed stages of disease other therapy options like radiochemotherapy and immunotherapy gained importance. Based on the fact that NRP2 and its ligand VEGF-C predicted treatment response to radiochemotherapy in patients (7), we analyzed the response of our KO and WT cell lines to radiotherapy and combined radiochemotherapy with cisplatin (Figures 7A,B and Supplementary Figure 11). Significances for plating efficacy are shown in Supplementary Figure 12, and the alpha–beta ratio defined from interpolation of linear–quadratic cell survival curves are shown in Supplementary Figures 13A,B. Our results indicate that there was no immediately visible effect between both KOs and their parental WT cell line. All cell lines responded to additional cisplatin treatment with significantly reduced clonogenic survival and plating efficacy. To identify the potential benefit of chemotherapy in addition to radiation treatment, we calculated the radiobiological enhancement ratio (RER) for each subset. The RER is an indicator of the radiosensitizing effect of any potential agent used as it compares the ratio of the surviving fraction from the radiation only to the radiation in combination with any agent at a specific dose. Analysis of RER showed a higher benefit of radiochemotherapy for KO cells than for WT cells (Figure 7C and Supplementary Figure 13C).

## Discussion

### NRP2 and GLI2 Interplay Is Dependent on the Ligand

The close relationship of NRP2 to GLI2 was discovered by correlation of genes in a provisional TCGA BCa data set (Supplementary Table 1A). Interestingly, this correlation of GLI2 and NRP2 was even stronger (*r* = 0.709) than the correlation of GLI2 to either GLI1 (*r* = 0.555) or GLI3 (*r* = 0.252). This is surprising because GLI1 is known to be a direct target gene of GLI2 in both the canonical and non-canonical Hh pathway (14, 16, 29, 30) (Supplementary Table 1B). We analyzed GLI1 levels for additional functional verification of GLI2 knockdown and could confirm that GLI1 levels were reduced following knockdown with a GLI2-specific siRNA pool. Since GLI1 levels are directly dependent on the expression of GLI2, we cannot fully exclude the possibility that changes in NRP2 could also be mediated through GLI1 or other downstream targets of GLI2 despite the fact that correlation between NRP2 and GLI2 is by far highest compared to GLI1 or GLI3 in a patient cohort. Whether or not this interdependency of the two gene products is a direct or indirect effect was demonstrated in two human BCa cell lines that GLI2 levels are regulated in part through NRP2 and that NRP2 mRNA levels are partially regulated by GLI2 signaling in a TGFβ1-dependent manner. In contrast to cell lines J82 and HS853T, another cell line (5637) showed no changes in the expression of these genes possibly because TGFβ1 failed to induce their expression. Consequently, the relationship of NRP2 and GLI2 could not be observed in this cell line (Supplementary Figure 14). Because prediction of overall and disease-free survival could be improved by combining NRP2 and GLI2 gene expression in the TCGA patient cohort, these results suggest a functional interplay between NRP2 and GLI2 in regulating tumor growth, although the mechanisms of this interplay still remain elusive and merit further investigation. Notably, GLI2 knockdown also changed the ratio of isoforms NRP2a and NRP2b in favor of the latter in two BCa cell lines. It was recently described in a lung cancer model that while NRP2a is almost dispensable for tumor formation and metastasis, NRP2b severely impacted these traits (31). The authors demonstrated that TGFβ1 predominantly upregulated NRP2b and that TGFβ1-dependent stabilization was specifically dedicated to isoform NRP2b. In our model, both NRP2 isoforms were equally increased on mRNA level, but this induction might be the result of compensating for increased protein degradation following TGFβ1 treatment given that the protein level of total NRP2 was not significantly increased.

### NRP2 Positively Regulates Osteopontin Expression

The active ligand TGFβ1 is a potent inducer of EMT. For investigating how NRP2 might enhance EMT, we chose a qPCR array containing 84 genes involved in EMT signaling and checked cDNA from two NRP2 knockout cell lines and their parental wild-type cell line RT112. Using this approach, a number of genes were deregulated in both KO clones compared to the parental cell line irrespective of the housekeeping gene used. Validation of these genes in four independent biological repeats confirmed their altered expression, although the change for CALD1, CDH2, and SPARC1 was not significant. Because the expression of CDH2 mRNA was sufficient to detect this target by Western blot, we checked the expression of the corresponding protein N-Cadherin as well as its counterpart E-Cadherin as control in treated and untreated conditions. Although N-Cadherin expression doubled in KO clones without TGFβ1-treatment, only one KO clone showed a significant increase. However, when TGFβ1-treatment was applied, we detected a significant reduction in both KO clones, although the change was less dramatic than for N-Cadherin in the untreated condition. There have been numerous reports in the past stating the significance of cadherin switching in progression and malignancy of BCa pointing to the importance of elucidating the mechanism for targeted therapy (32–37). Moreover, it was already shown that NRP2 and E-Cadherin expression are connected in multiple cancer types (23, 38–41). However, unlike the positive correlation in our model system, all publications reported a negative correlation. It has previously been described for melanoma that Osteonectin (gene SPARC) can downregulate E-Cadherin (42). We could show a 4.5-fold (KO #2) to 9.8-fold (KO #1) upregulation of SPARC compared to WT when treated with TGFβ1. Although this change was not found to be significant, it was still the highest deregulated gene within the panel. Therefore, the reduced E-Cadherin (CDH1) expression might be a direct result of increased SPARC expression. Given that the authors of the cited paper only investigated E-Cadherin by Western blot but not by qPCR and the fact that the change of E-Cadherin in our model was only visible on protein level suggests that SPARC might not control E-Cadherin transcriptionally. Previous studies showed that SPARC induces β-catenin nuclear localization and binding to the transcriptional regulator lymphocyte-enhancer factor-1 (LEF-1) (43, 44), whereas E-Cadherin forms alternative complexes with β-catenin in the adherens junctions (AJ). These AJ complexes prevent β-catenin nuclear localization and transactivation as well as E-Cadherin internalization (45). We can hypothesize that SPARC can induce loss of β-Catenin in the AJ by triggering its nuclear translocation that might result in E-Cadherin endocytosis and degradation. Of note, protein levels of N-Cadherin (CDH2) after TGFβ1-treatment did not change dramatically anymore, potentially indicating that this change is independent of the ligand TGFβ1.

In order to investigate if the identified targets were specific to that cell line or if signaling pathways were deregulated for compensation of complete NRP2 loss in the knockout cells, we used siRNA-mediated depletion of NRP2 in another human cell line (J82). The results confirmed deregulation of all but one gene (STEAP1), but the direction of change was only consistent for one gene (SPP1/Osteopontin/OPN). Thus, changes in other genes are either cell line specific or long-term KO models adapt to missing NRP2 by deregulation of other EMT pathways that were initially downregulated in the short-term knockdown model (for example, CALD1, CDH2, and SPARC). But given that Osteopontin was the only target significantly downregulated in both NRP2 KO and knockdown models in different cell lines, we propose that this dependency might be a general mechanism. To our knowledge, this is the first report linking NRP2 and SPP1/OPN in any tissue or cancer entity by showing that NRP2 acts upstream of SPP1 in a TGFβ1-independent manner. This can be an explanation for the VEGF-induced OPN expression, which was demonstrated in a large number of cases (25). OPN was shown to be upregulated in multiple cancer types including breast and prostate cancer as well as glioblastoma and melanoma (46, 47). Regarding BCa, immunohistochemical staining of OPN demonstrated significant correlation with tumor stage (27). More recently, Wong and colleagues showed that OPN expression correlates with disease stage and grading and that higher OPN expression led to decreased survival in multiple patient cohorts (26). Of note, we could not see the same in our TCGA data set when applying median expression for overall and disease-free survival (Supplementary Figure 9). However, combining NRP2 and OPN expression slightly improved prediction of disease-free but not overall survival (Supplementary Figure 10). Since OPN is a secreted soluble molecule, it may serve as an attractive non-invasive prognostic marker in serum or urine. One study investigated plasma OPN levels before and after tumor resection in 50 patients with BCa and found significantly higher preoperative OPN levels in patients with muscle invasive tumors despite the relatively low number of patients. OPN levels also increased significantly with T stage when patients had undergone radical cystectomy. The strong trend of correlation with tumor grade and predicting recurrence did not reach statistical significance, potentially indicating that patient number for this analysis was too low (48). Similar to OPN, the expression pattern of NRP2 was previously reported to be significantly associated with pathological stage and tumor grade in a BCa cohort, suggesting a prominent role of NRP2 in BCa progression (49).

Because OPN is also associated with bone matrix formation, it would be highly interesting to analyze if the connection between NRP2 and OPN is also true for cancer entities like breast and prostate cancer, where bone metastases remain a big challenge.

### NRP2 as Target for Radiochemotherapy

Overall and disease-free survival data suggested NRP2 as prognostic indicator for a TCGA BCa cohort. Our group previously showed that expression of NRP2 as well as its ligand VEGF-C could predict treatment outcome of BCa patients following TURBT and radiochemotherapy. This clinical finding prompted us to use our NRP2 KO and WT cell lines for an *in vitro* assay to determine their clonogenicity after radio(chemo)therapeutic treatment. When looking at the radiotherapy group only, no significant differences between both KO and the WT cell lines were found, which confirmed the previous finding that NRP2 expression alone in the patient group receiving only local radiotherapy was not a prognostic factor (7). When the same analysis was applied to the group of patients, which received radiochemotherapy, NRP2 was highly prognostic for overall and cancer-specific survival. In accordance to this clinical observation, we revealed a radiation dose-dependent trend toward higher profit of additional chemotherapy in KO cells, suggesting that NRP2 downregulation results in BCa radiochemosensitization. The exact role of NRP2 for radiochemotherapy in BCa warrants further investigation using additional cell lines and animal models.

Taken together, our study demonstrated that mRNA expression of NRP2 and GLI2 highly correlate in BCa cell lines and the TCGA BCa cohort. They influence each other's expression depending on the presence or absence of TGFβ1, a potent inducer of EMT. Moreover, screening of 84 genes involved in EMT identified SPP1/Osteopontin as a downstream target of NRP2 in two different BCa cell lines using different model systems.

Future research is needed to evaluate the exact mechanism of how NRP2 and GLI2 communicate bidirectionally, how NRP2 modulates SPP1 transcription, and what implications this will have for development and progression of other cancer entities apart from bladder carcinoma.

## Materials and Methods

### Cell Lines and Cell Culture

The cell line RT112 (DSMZ) was maintained in MEM alpha medium with GlutaMAX supplemented with 10% FBS (fetal bovine serum, both Gibco, Life Technologies, Waltham, USA). The cell line and their knockout derivatives were validated to be RT112 by single nucleotide polymorphism (SNP) profiling (performed by Multiplexion GmbH, Friedrichshafen, Germany). Cell line J82 (ATCC) and HS853T (ATCC) were cultured in DMEM medium (4.5 g/L glucose) supplemented with 10% FBS (both Gibco, Life Technologies, Waltham, USA), 1% HEPES solution, and 1% MEM non-essential amino acids (both Sigma-Aldrich, St. Louis, USA). The cell line 5637 (ATCC) was cultured in RPMI supplemented with 10% FBS (both Gibco, Life Technologies, Waltham, USA). All cell lines were subject to regular testing for excluding mycoplasma contamination (last on 2nd April 2019). All cells were maintained at standard conditions in a humidified incubator with 5% CO_2_ at 37°C.

### CRISPR/Cas9-Mediated Deletion of NRP2 in RT112 Cells

Plasmid-based CRISPR/Cas9 technology was used to generate RT112 cell clones deficient in NRP2 expression according to the protocol established by Ran et al. (50). In brief, DNA double stranded oligonucleotides located in the exon 3 splice acceptor region (NRP2e3gu1-top: 5′-CACCGGATAAAGTCATACCTGGGTG-3′, NRP2e3gu1-bottom: 5′-AAACCACCCAGGTATGACTTTATCC-3′) and exon 3 coding region (NRP2e3gu2-top sequence: 5′-CCACCGGGTGAACTTGATGTAGAGCA-3′; NRP2e3gu1-bottom sequence: 5′-AAACTGCTCTACATCAAGTTCACCC-3′) of the NRP2 locus were designed using the Benchling software (San Francisco, USA) and cloned into pSpCas9 BB2A-GFP for gu1 (PX458, Addgene, LGC Standards, UK) or pSpCas9 BB-2A-Puro for gu2 (PX459v2, Addgene). RT112 cells were transfected by calcium phosphate precipitation with a 1:1 ratio of PX458-NRP2e3gu1 and PX459v2-NRP2e3-gu2 for 8 h, kept under puromycin selection (0.5 μg/ml) for 24 h and then seeded at low density. One hundred clones were picked after two weeks, grown and checked for the desired 110 bp deletion in NRP2 exon 3 by PCR on isolated genomic DNA using primers 5′-AGTGCCCTTCGCTTATCCATC-3′ and 5′-TCTAAGACGCCCATCTCCCG-3′. Clones that carried the deletion in the NRP2 locus were further checked for mutations within the corresponding region of the NRP1 locus (primer sequences 5′-GCTGGATGATGCTGGTGTCTA-3′ and 5′-TTCTACCGTAAGCTGTTCACTC-3′) and for Cas9 (primer sequences 5′-CGACGACAGCCTGACCTTTA-3′ and 5′-TTGATGCCCTCTTCGATCCG-3′) to exclude integration of transfected plasmids. Sanger sequencing of the PCR amplification products verified the deletions. TOPO cloning of DNRP2 PCR products and subsequent Sanger sequencing of single mutated alleles yielded the sequences of individual deletion alleles for NRP2 in two individual RT112 DNRP2 cell clones. Three independent mutant alleles were identified for both RT112 DNRP2 clones, with altered exon 3 splice acceptor sequences and/or introduction of frame shifts due to nucleotide insertions or deletions:

(clone: mutant alleles; mutation; location of mutation in NRP2 exon 3)
#J9 (KO#1): J9high-3 delAG; delCT delAG (−2;−1); delCT (107; 108)
#J9 (KO#1): J9high-2 del111 deletion: −4 to +107
#J9 (KO#1): J9high-1 del111 deletion: −2 to +109
#J32(KO′2): #J32-5 insC; delT insC (−3); delT (107)
#J9 (KO#2): #J32low-1 del110 deletion: −3 to +107
#J9 (KO#1): #J32-P2 del111 deletion: −4 to +107

Both RT112 DNRP2 cell clones were checked for the absence of NRP2 expression by Western blot immunostaining using an anti-NRP2 antibody (R&D Systems, Minneapolis, USA). For reasons of simplicity, the clones are referred to as knockout (KO) #1 (clone #J9) and KO #2 (clone #J32) in this publication.

### TGFβ1-Induced EMT

RT112 WT and derived KO cell lines were seeded at 1 × 10^5^ cells per well in a 6-well culture plate containing 2 ml of serum-reduced (5 % FBS) growth medium either including or lacking 5 ng/ml TGFβ1 (Miltenyi Biotec, Bergisch Gladbach, Germany). After 48 h in the incubator, the medium was renewed and cells were incubated for another 24 h before RNA or protein isolation. In total, four independent biological repeats were performed with cells at different passages. RNA of two repeats was used for “RT^2^ Profiler PCR Array” for human EMT and all four repeats were used for validation of identified targets by qPCR. Protein was used for immunoblotting.

### Transfection of Cell Lines With siRNA

Cell lines J82, HS853T, and 5637 were used for knockdown experiments. 2 × 10^5^ (J82, HS853) or 5 × 10^5^ (5,637) cells per well were seeded in a 6-well culture plate with 2 ml complete growth medium (10% FBS) and incubated 24 h to allow attachment. Next, medium was renewed and liposomal transfection was conducted according to the manufacturer's protocol using 12 μl of Lipofectamine RNAi MAX (Thermo Fisher Scientific, Waltham, USA) and 50 nM siRNA (SMARTpool by Dharmacon, Lafayette, USA). The catalog number of siRNA pools was D-001810-10-20 (siSCR, control), L-017721-00-0010 (siNRP2), L-0066468-00-0005 (siGLI2), and L-012558-00-0005 (SPP1). Cells were treated with TGFβ1 as described above.

### RNA Isolation, cDNA Synthesis, and qPCR

RNA was isolated with the RNeasy Plus Mini Kit (Qiagen, Hilden, Germany) by adding 350 μl of lysis buffer RLT Plus supplemented with 1% β-mercaptoethanol (Sigma-Aldrich, St. Louis, USA) to a well of a 6-well plate that was previously rinsed with PBS. Using a scraper, lysed cells were collected from the plate and transferred to a DNA removal column. The following steps were carried out according to the manufacturer's protocol and RNA was eluted from the column by addition of 30 μl of RNase-free water (Qiagen, Hilden, Germany). cDNA from 1,000 ng of total RNA input was synthesized by employing the PrimeScriptTM RT Reagent Kit (Takara Bio Inc., Kusatsu, Japan) according to the manufacturer's instructions. cDNA was diluted 1:5 with RNase-free water before continuing with real-time quantitative polymerase chain reaction (qPCR). qPCR was conducted using the TB GreenTM Premix Ex TaqTM II (Takara Bio Inc., Kusatsu, Japan) according to the manufacturer's protocol for a total reaction volume of 20 μl. The qPCR cycling conditions were set on a StepOnePlus system (Applied Biosystems, Waltham, USA): 94°C for 3 min, 40 cycles: 94°C for 15 s, 58°C 60 s, 72°C 60 s followed by a melt curve to 95°C in steps of 0.3°C. All experiments were conducted using at least two (for housekeeping genes) or three technical replicates (other targets) and most experiments included three different housekeeping genes as control: ACTB, GAPDH, and HPRT1. All primer sequences are listed in Supplementary Table 3. cDNA of the following BCa cell lines was used for qPCR analyses of NRP2 and GLI2: 5637 (ATCC), 639V (DSMZ), Cal29 (DSMZ), EJ28 (University Frankfurt), HS853T (ATCC), HT1376 (ATCC), J82 (ATCC), UMUC-3 (ATCC), UMUC-14 (Sigma-Aldrich), UMUC-16 (Sigma-Aldrich), VMCUB (DSMZ), KU1919 (DSMZ), RT112 (DSMZ), T24 (DSMZ), and TCC-SUP (DSMZ). HPRT1 served as housekeeping gene. ΔCt values were used to calculate correlation in SUMO software (http://angiogenesis.dkfz.de/oncoexpress/software/sumo/).

### RT^2^ Profiler PCR Array for Human EMT

cDNA synthesis was performed following RNA isolation as described above (total volume: 10 μl). For each 96-well plate, a master mix was prepared composed of 1,050 μl of TB Green^TM^ Premix Ex Taq^TM^ II, 42 μl of ROX dye, 1,000 μl of RNase-free water, and 10 μl of cDNA sample. From this master mix, 20 μl was added to each well. The PCR program was identical to the one described above. For the human EMT PCR array (Qiagen, Hilden, Germany), two out of four biological replicates were chosen that displayed values closest to the median. Later, all four biological replicates were evaluated for target validation. Data were extracted using the housekeeping gene HPRT1 from the EMT profiler plate.

### Protein Isolation and Immunoblotting

Protein was isolated from 1-well of a 6-well plate by washing cells once with PBS before adding cold 200-μl RIPA buffer (Thermo Fisher Scientific, Waltham, USA) supplemented with Complete inhibitor (Roche, Basel, Switzerland), proteinase, and phosphatase inhibitor (both Thermo Fisher Scientific, Waltham, USA). Lysates were collected with cell scrapers, transferred to a 1.5-ml reaction tube and centrifuged at 10,000 rpm at 4°C for 10 min in a 5415R cooling centrifuge (Eppendorf, Hamburg, Germany). Supernatant was transferred to a new reaction tube and protein was quantified by Pierce^TM^ BCA Protein Assay Kit (Thermo Fisher Scientific, Waltham, USA) according to the manufacturer's protocol. Thirty-five micrograms of total protein lysate was loaded into each pocket of a 12-well Bolt 4–12% Bis-Tris Plus Gel (Thermo Fisher Scientific, Waltham, USA) running in an XCell SureLock^TM^ Electrophoresis Cell (Invitrogen, Carlsbad, USA) at 90 V for 2 h in MOPS buffer (Thermo Fisher Scientific, Waltham, USA). Wet transfer to a methanol-activated 0.2-μm Amersham^TM^ Hybond^TM^ Low Fluorescence PVDF membrane (GE Healthcare, Chicago, USA) was achieved by applying 90 V for 4 h in a cooled Mini-PROTEAN® three transfer tank (Bio-Rad, Hercules, USA). Membranes were washed once with TBS-T before blocking the membrane for 1 h at room temperature with 2.5% ECL Prime^TM^ blocking agent (GE Healthcare, Chicago, USA) dissolved in TBS. Membranes were incubated at 4°C overnight with primary antibodies (see Supplementary Table 4 for the complete list of used antibodies) diluted in 2.5% ECL Prime^TM^ blocking agent solution before applying three washing steps with TBS-T. Then, membranes were incubated with the appropriate secondary antibodies diluted in 2.5% ECL Prime^TM^ blocking agent solution for 1 h at room temperature. Following another three washing steps with TBS-T, chemiluminescent detection was performed by first incubating the membrane with Pierce® ECL Western Blotting Substrate (Thermo Fisher Scientific, Waltham, USA) and subsequent detection of the signal in auto-rapid mode in a ChemiDoc^TM^ MP Imaging System (Bio-Rad, Hercules, USA). Colorimetric images were taken for determining the molecular weight of the signals. Calculation of optical densitometry was performed with ImageJ software.

### Colony Forming Assay Following Radiochemotherapy

For radiochemotherapy treatment, cells were seeded into a 6-well plate at a density of 4 × 10^5^ cells per well and treated with cisplatin (TEVA GmbH, Ulm, Germany) for 24 h at concentrations of 1.52 × 10^−6^ M that corresponded to the IC_5_ value of KO #2 (Supplementary Figure 11). Twenty-four hours after start of the treatment, cells were trypsinized and used for radiobiological colony forming assay. Clonogenic survival was determined by seeding of 500 (ionizing radiation—IR only) or 750 (IR + cisplatin) cells in technical triplicates into 6-well plates containing 2 ml of complete growth medium. Cells were cultivated overnight and then irradiated with doses of 0, 2, 4, 6, and 8 Gy (Yxlon Y.TU 320; 200 kV X-rays, dose rate 1.3 Gy/min at 20 mA, filtered with 0.5 mm Cu). Irradiated cells were returned to the incubator for allowing recovery and growth for 6 days. Colonies were fixed by the addition of 600 μl of 37% formaldehyde solution (Merck, Darmstadt, Germany) directly to the culture medium and incubation at room temperature for 30 min. Following removal of this solution and a washing step with normal tap water, 1 ml of a 0.05% crystal violet solution (Sigma-Aldrich, St. Louis, USA) was added to each well for 30 min at room temperature for staining colonies. Crystal violet was removed, and wells were washed twice with normal tap water and dried overnight before manually counting colonies using a stereomicroscope (Zeiss, Oberkochen, Germany). Cell survival data were entered into the SPSS program for calculation of α and β values, curve fitting to the linear quadratic model, and determination of statistical significance.

### Analysis of the TCGA Patient Cohort Data

From the TCGA patient cohort data set, Pearson coefficient was determined using SUMO software and significance was calculated by two-tailed, unpaired Student's *t* test. For evaluation of combined NRP2/GLI2 signature expression, the data for each of these genes were normalized to median across the entire dataset and log2 transformed. Further, the mean of two genes was calculated for each patient and the subset with known survival data was extracted from complete cohort. Finally, the up- and down-regulated groups for Kaplan–Meier analysis were defined as the mean of NRP2/GLI2 expression was positive or negative value accordingly.

### Statistical Analysis

The cell survival curves were analyzed using the Statistical Package for the Social Sciences (SPSS) v23 software as described previously (51) by linear–quadratic formula S(D)/S(0) = exp−(αD + βD2) using stratified linear regression. A *p* < 0.05 was considered statistically significant. Correlation of gene expression levels was evaluated by SUMO software using Pearson correlation coefficient. IC_50_ and IC_5_ values (50 and 5% inhibitory concentration) were determined by non-linear regression using GraphPad Prism software (San Diego, USA).

## Data Availability Statement

All datasets generated for this study are included in the article/Supplementary Material.

## Author Contributions

AS performed most experiments and wrote and edited the manuscript. IG performed revision experiments and edited the manuscript. RB helped to teach CRISPR/Cas9 technology and designed the guide RNAs. SFu and KE provided cell lines and cDNA of cell lines and edited the manuscript. SFo and KD edited the manuscript. TM generated and validated the knockout clones from cell line RT112. TM, AD and MM supervised and guided AS, edited the manuscript, and aided in scientific questions including experimental design. AD helped perform radiochemotherapy and colony forming assay.

### Conflict of Interest

The authors declare that the research was conducted in the absence of any commercial or financial relationships that could be construed as a potential conflict of interest.
