# Supplementary material for: Linking NRP2 With EMT and Chemoradioresistance in Bladder Cancer
Source: Front Oncol. 2020 Jan 21;9:1461. doi: 10.3389/fonc.2019.01461 (PMC6986262; doi:10.3389/fonc.2019.01461)
Supplement: Supplementary file 1 [file Data_Sheet_1.zip › Supplementary Table 3.DOCX]

| Gene | Forward 5’🡪3’ | Reverse 5’🡪3’ |
| --- | --- | --- |
| ACTB | ATGGAGTCCTGTGGCATCCA | AGTACTTGCGCTCAGGAGGA |
| CALD1 | TGGAGGTGAATGCCCAGAAC | GAAGGCGTTTTTGGCGTCTTT |
| CDH1 | GGCCTGAAGTGACTCGTAACG | GTTCAGGGAGCTCAGACTAG |
| CDH2 | CAGAATCAGTGGCGGAGATC | CCTTCTTCTTGGCGAATGATC |
| GAPDH | CAATGACCCCTTCATTGACC | TTGATTTTGGAGGGATCTCG |
| GLI1 | CAACTCGATGACCCCACCAC | TCTCTCTGGCTGGCCCATAA |
| GLI2 | TGGCTGACCTCAAGGAAGAT | CGTGGATGTGCTCGTTGTT |
| HPRT1 | TTGCGACCTTGACCATCTTTG | CTTTGCTGACCTGCTGGATTAC |
| NRP2 | ACCAGAACTGCGAGTGGATT | CGATGTTCCCACAGTGTTTG |
| NRP2a | CCCAGCTACGACATGGAGTA | GCTCCAGTCCACCTCGTATT |
| NRP2b | TCCGGAGAGATTGCCATTGA | GGCGGCCATTACGTAATACC |
| SPARC | AGCACCCCATTGACGGGTA | GGTCACAGGTCTCGAAAAAGC |
| SPP1 | GAAGTTTCGCAGACCTGACAT | GTATGCACCATTCAACTCCTCG |
| STEAP1 | CTATTCCATCTGTGAGTGACTC | GACAACAATTGGAAGGAAAACAGC |
| ZEB1 | GATTCTACACCGCCCAAAAA | AAGCGCTTTCCACATTTGTC |

Supplementary Table 3. Table of PCR Primers
