# Supplementary material for: Linking NRP2 With EMT and Chemoradioresistance in Bladder Cancer
Source: Front Oncol. 2020 Jan 21;9:1461. doi: 10.3389/fonc.2019.01461 (PMC6986262; doi:10.3389/fonc.2019.01461)
Supplement: Supplementary file 1 [file Data_Sheet_1.zip › Supplementary Table 4.DOCX]

Supplementary Table 4. Table of Antibodies

| Antibody Target  (Catalogue-Number) | Host | Company | Dilution |
| --- | --- | --- | --- |
| NRP2 (AF2215) | Goat | R&D Systems (Minneapolis, USA) | 1:400 |
| GLI2 (R770) | Rabbit | Cell Signaling (Danvers, USA) | 1:1000 |
| E-Cadherin (NB110-56937) | Rabbit | Novus Biologicals (Centennial, USA) | 1:1000 |
| N-Cadherin (610920) | Mouse | BD Biosciences (Franklin Lakes, USA) | 1:1000 |
| α-Tubulin (3873) | Mouse | Cell Signaling (Danvers, USA) | 1:1,000 |
| Goat, HRP-conjugated (305-035-003) | Rabbit | Jackson Immunoresearch (West Grove, USA) | 1:10,000 |
| Mouse, HRP-conjugated (NXA931) | Sheep | GE Healthcare (Chicago, USA) | 1:10,000 |
| Rabbit, HRP-conjugated (NA934) | Donkey | GE Healthcare (Chicago, USA) | 1:10,000 |
