# Supplementary material for: Linking NRP2 With EMT and Chemoradioresistance in Bladder Cancer
Source: Front Oncol. 2020 Jan 21;9:1461. doi: 10.3389/fonc.2019.01461 (PMC6986262; doi:10.3389/fonc.2019.01461)

Supplementary Figure 1

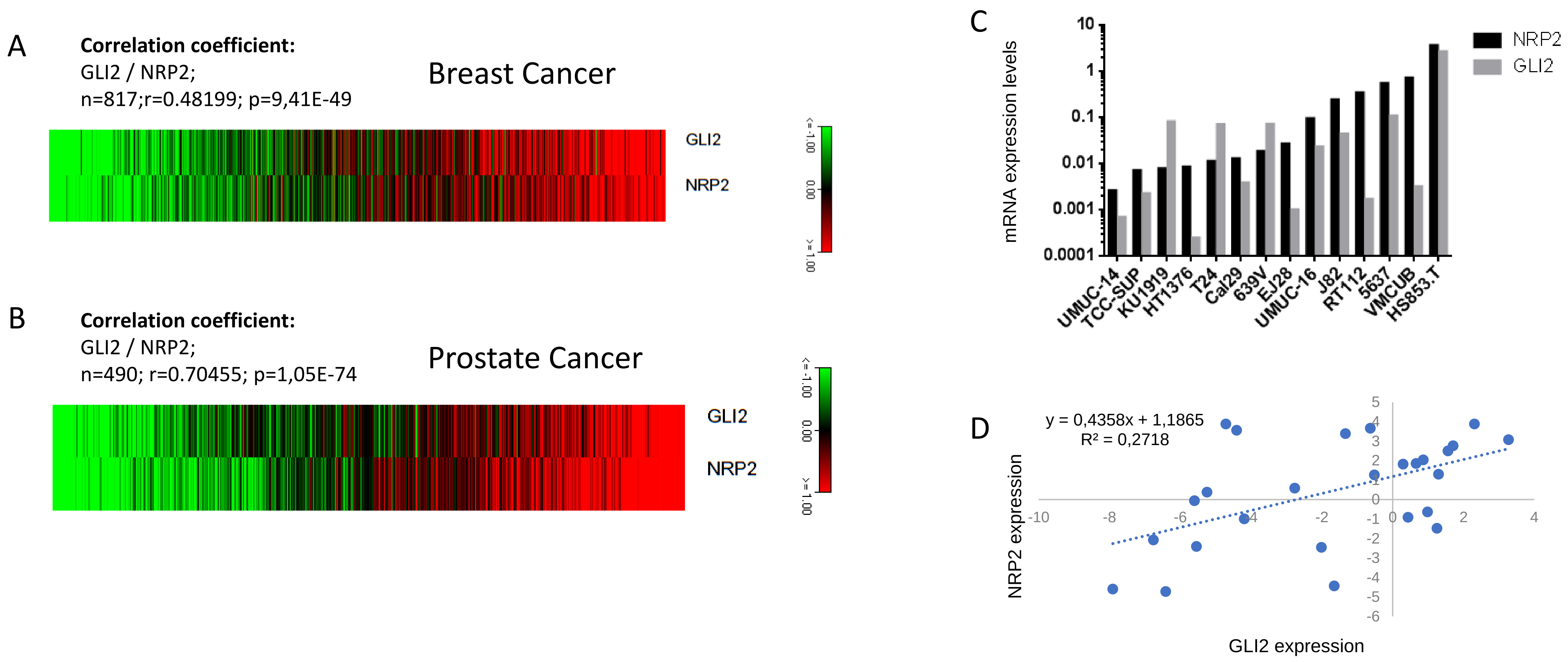

Supplementary Figure 2

A DFS BCa: NRP2

B DFS BCa: GLI2

C DFS BCa: NRP2 and GLI2

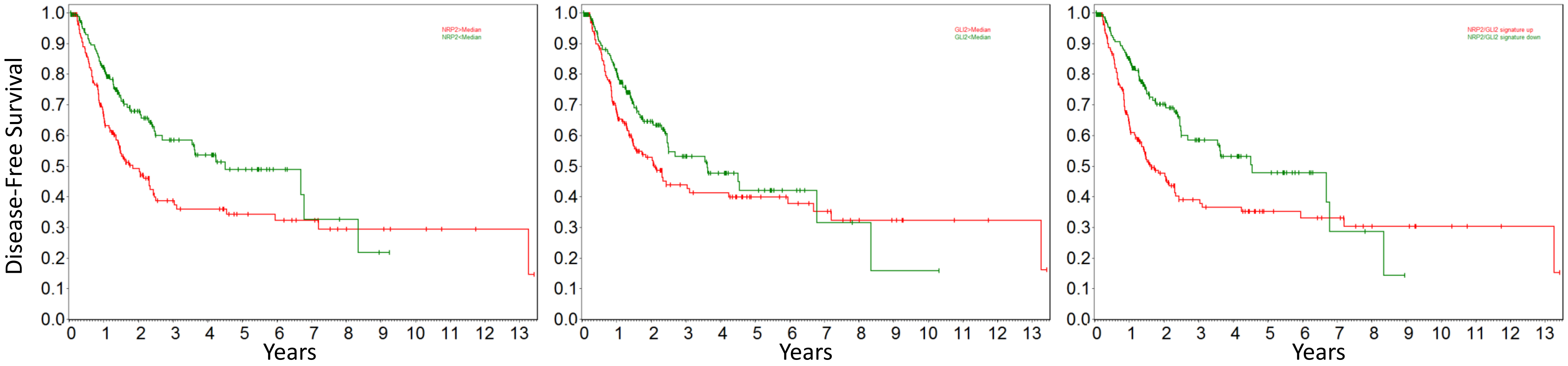

A

Individuals	154	164
Observed	83,00	58,00
Expected	65,89	75,11
Censored	71	106

Log-Rank=8.343
p=3,872E-003

B

Individuals	154	164
Observed	80,00	61,00
Expected	70,21	70,79
Censored	74	103

Log-Rank=2.717
p=9,925E-002

C

Individuals	157	161
Observed	87,00	54,00
Expected	67,57	73,43
Censored	70	107

Log-Rank=10.730
p=1,054E-003

Supplementary Figure 3

A

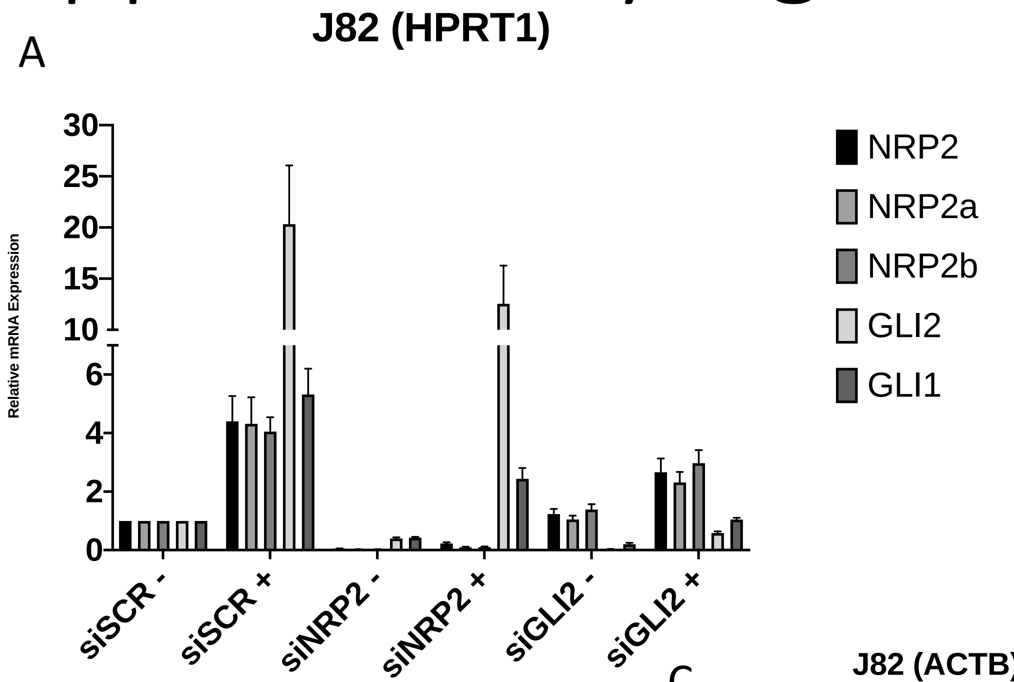

B

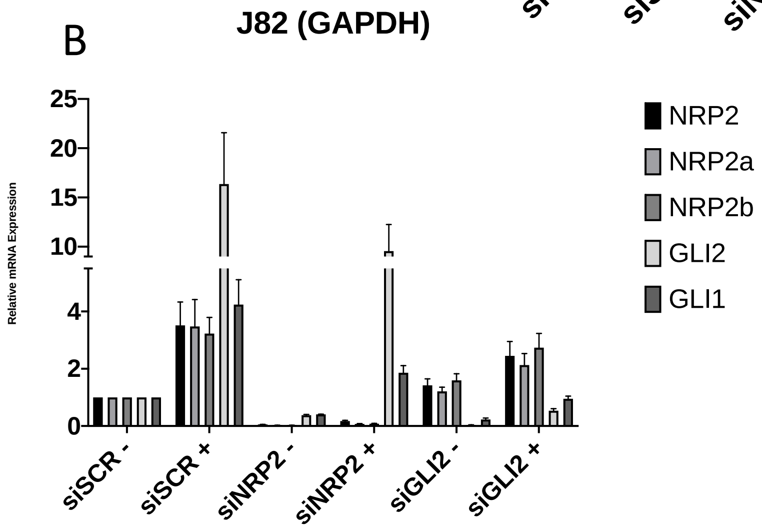

C

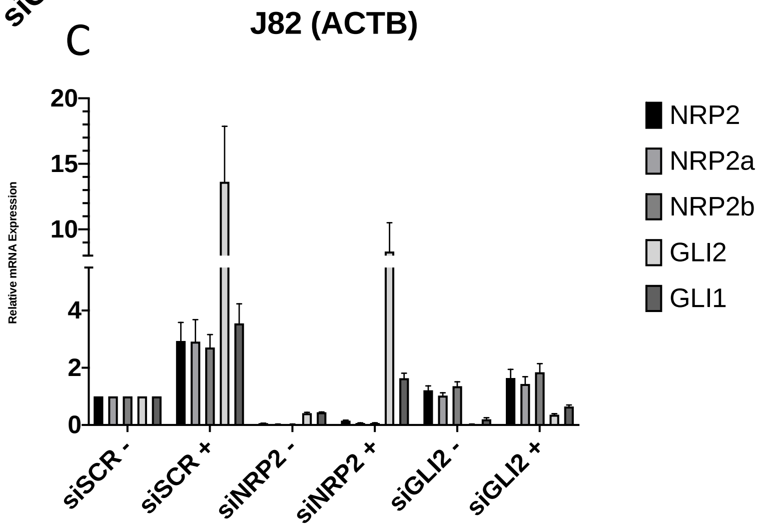

Supplementary Figure 4

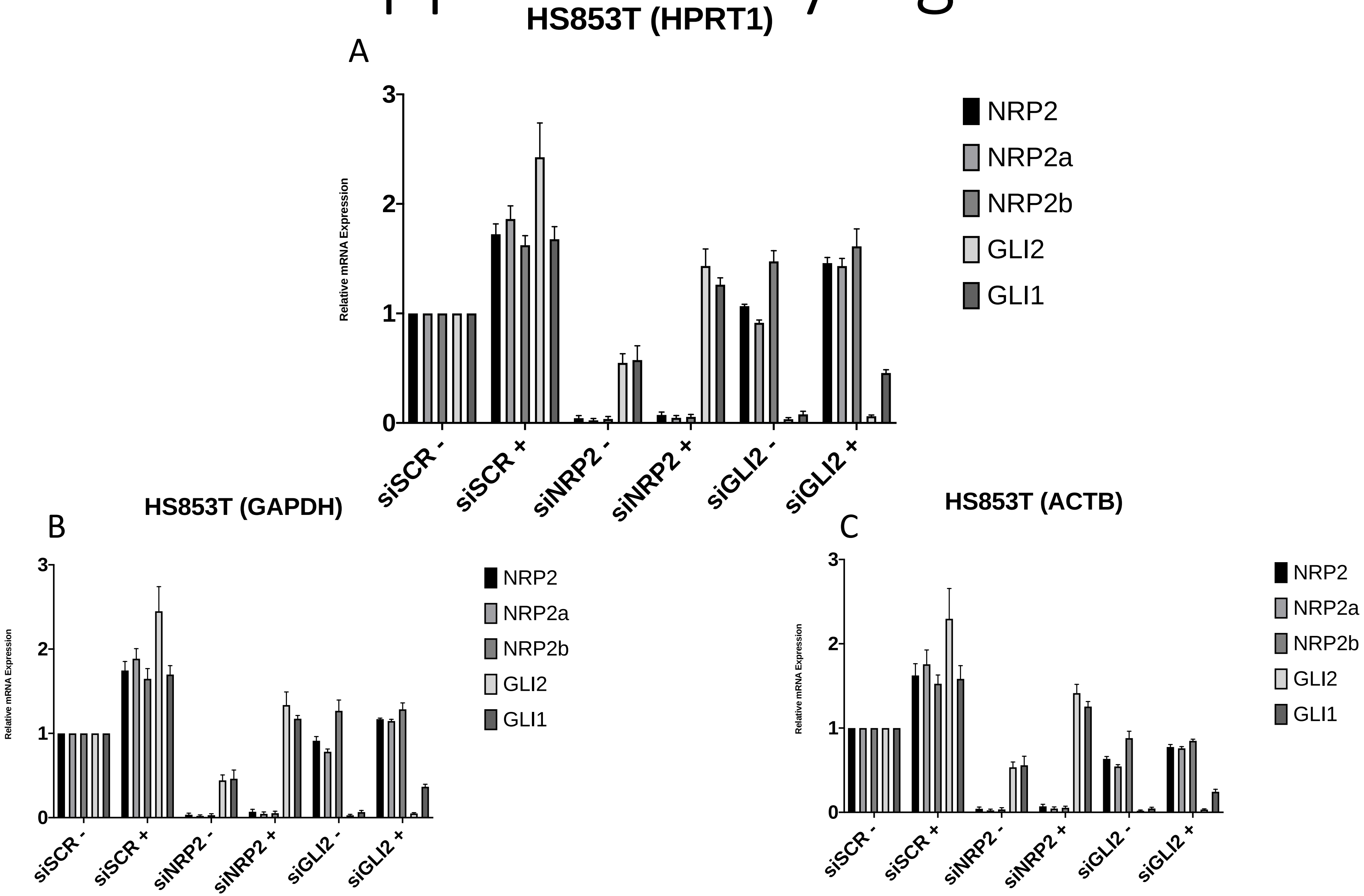

Supplementary Figure 5

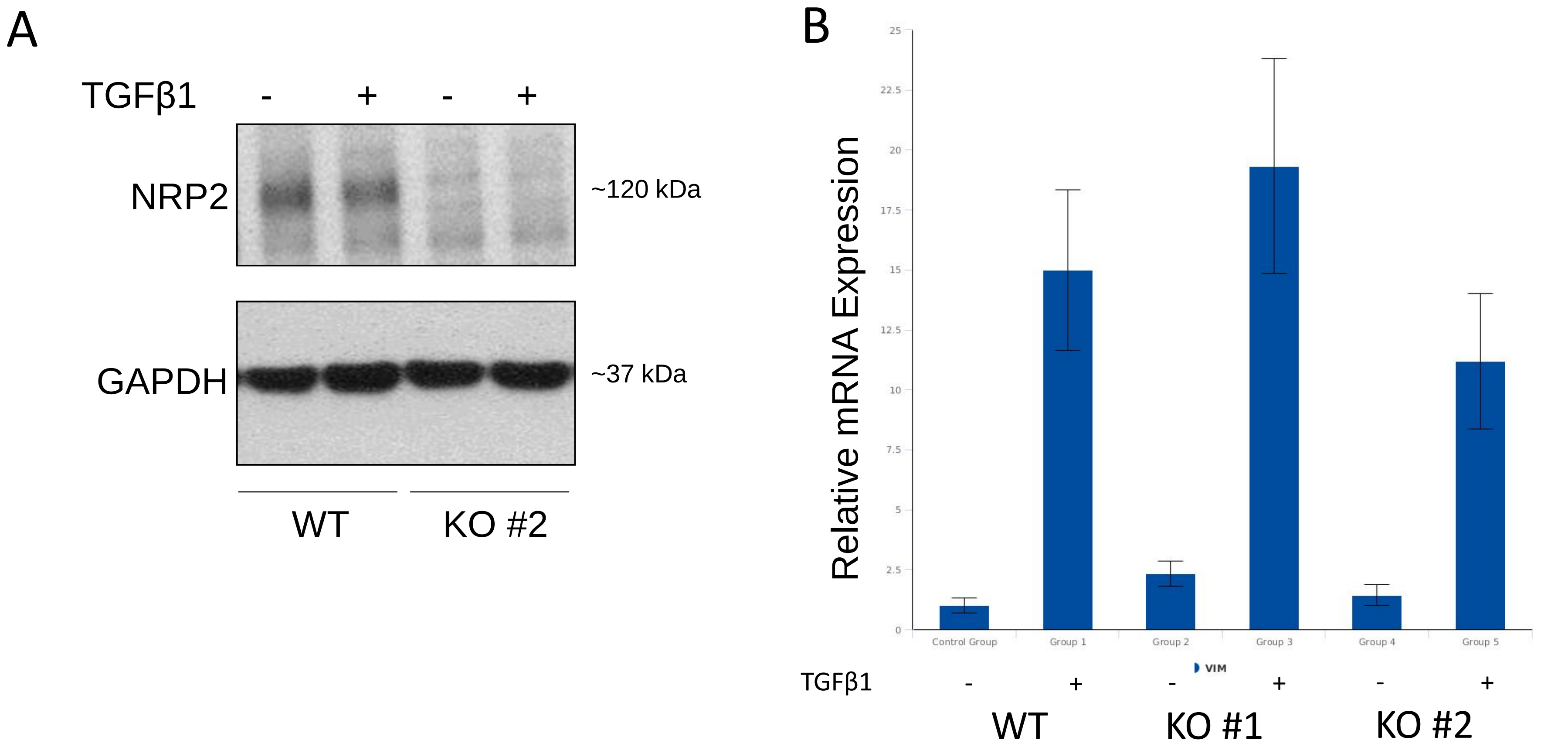

Supplementary Figure 6

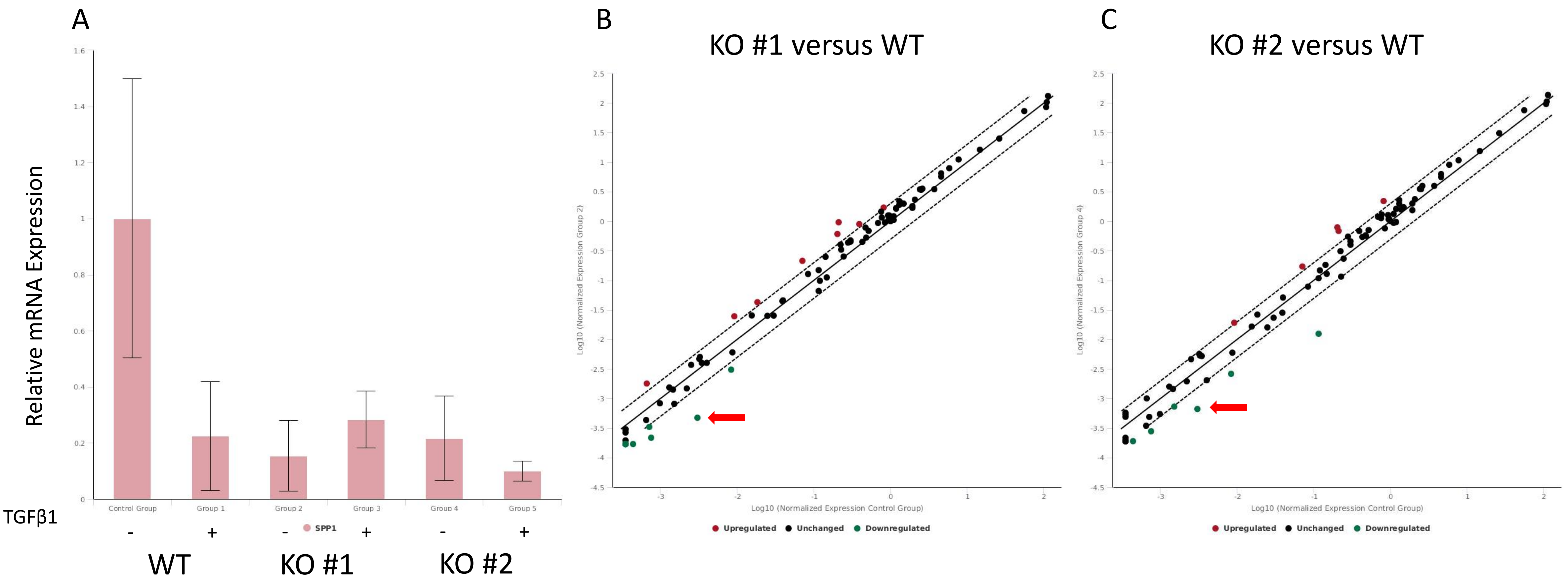

Supplementary Figure 7

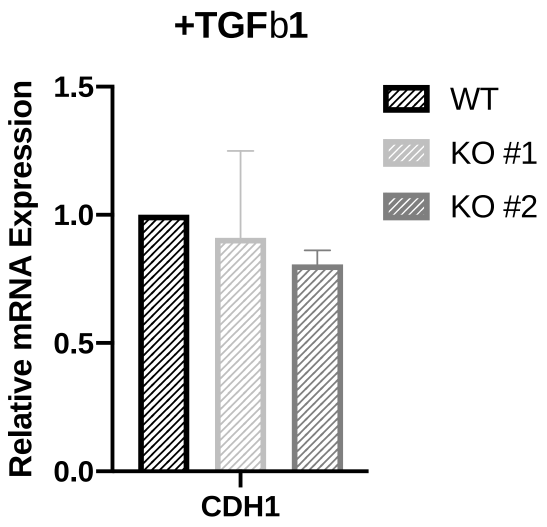

Supplementary Figure 8

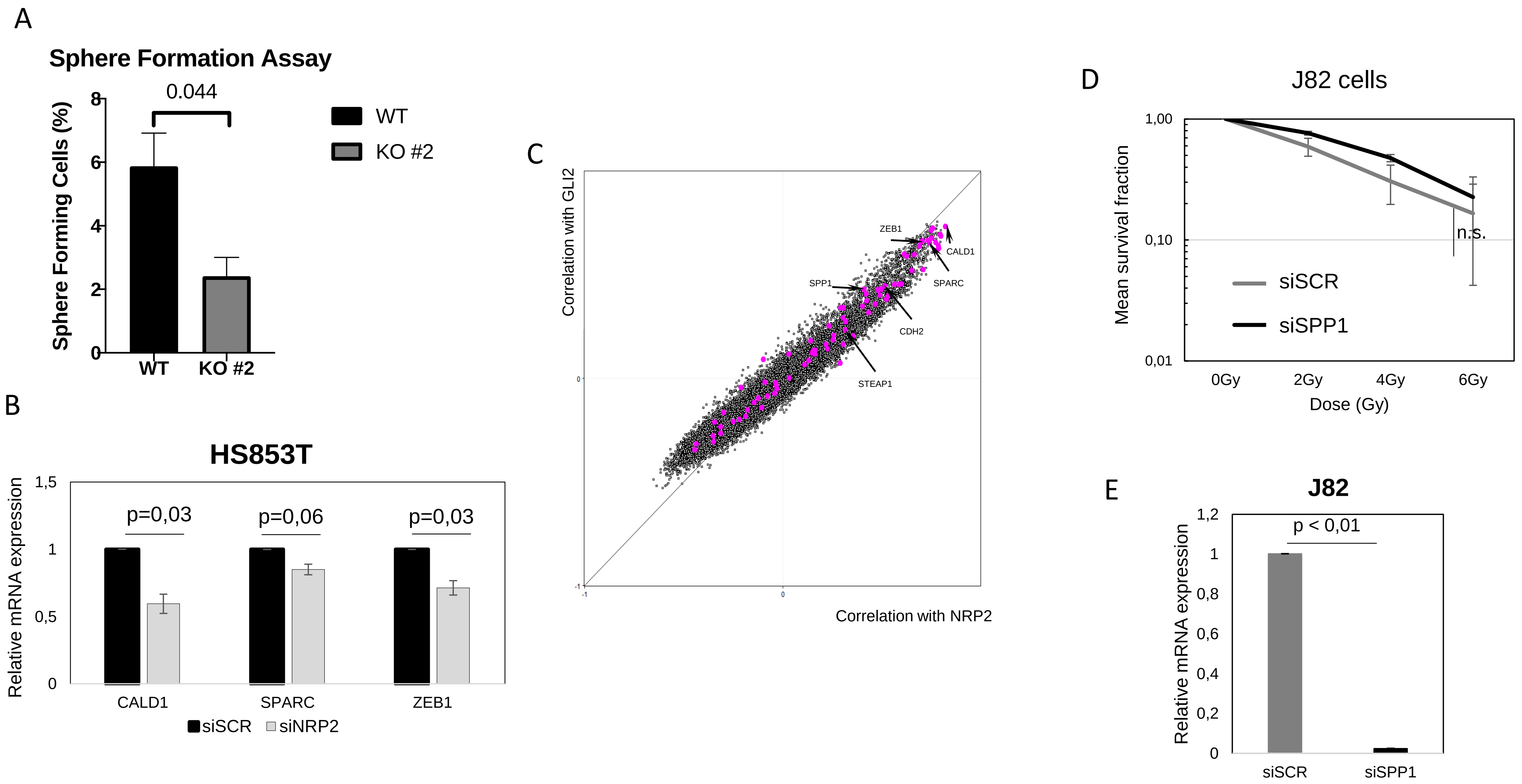

Supplementary Figure 9

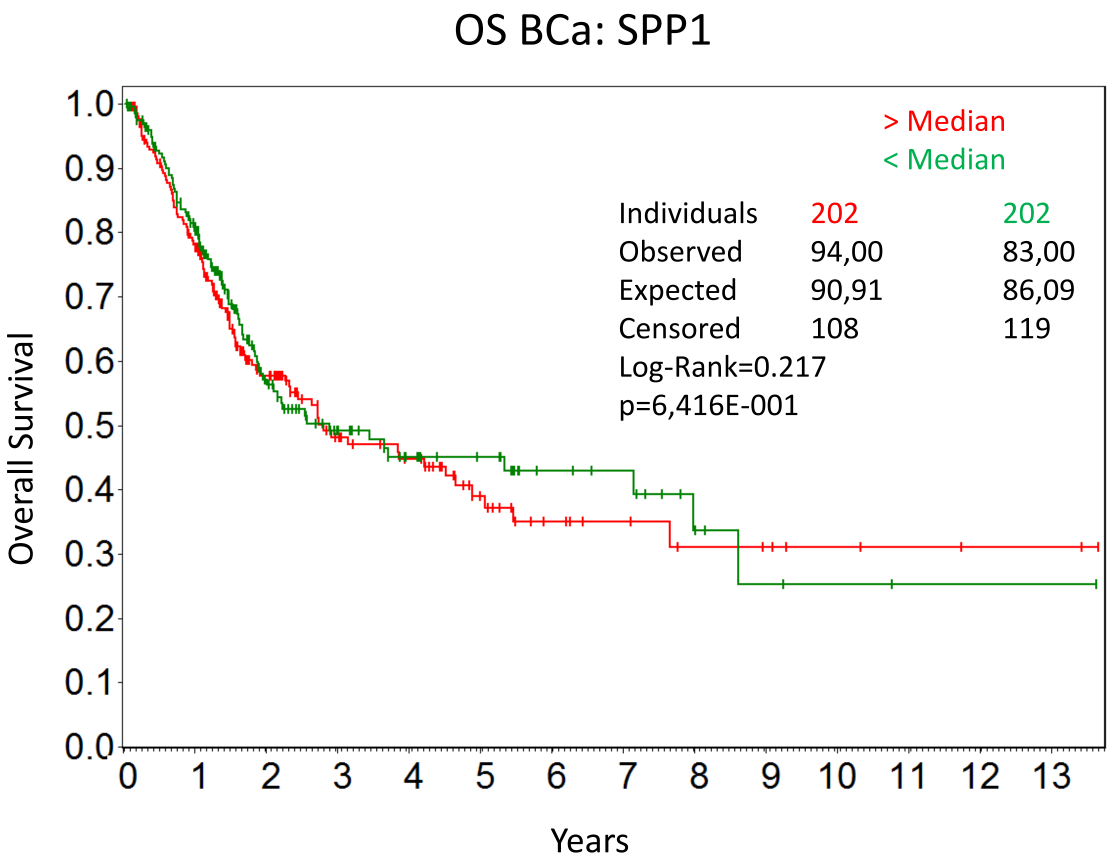

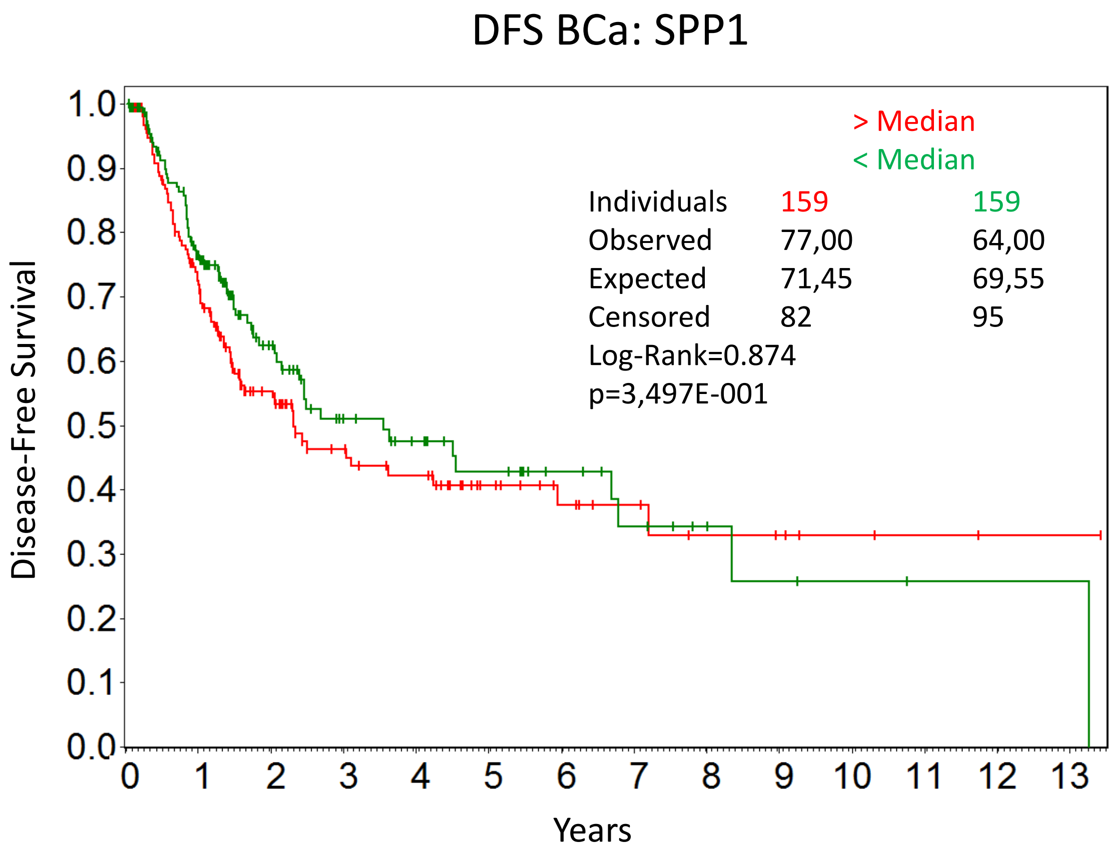

Supplementary Figure 10

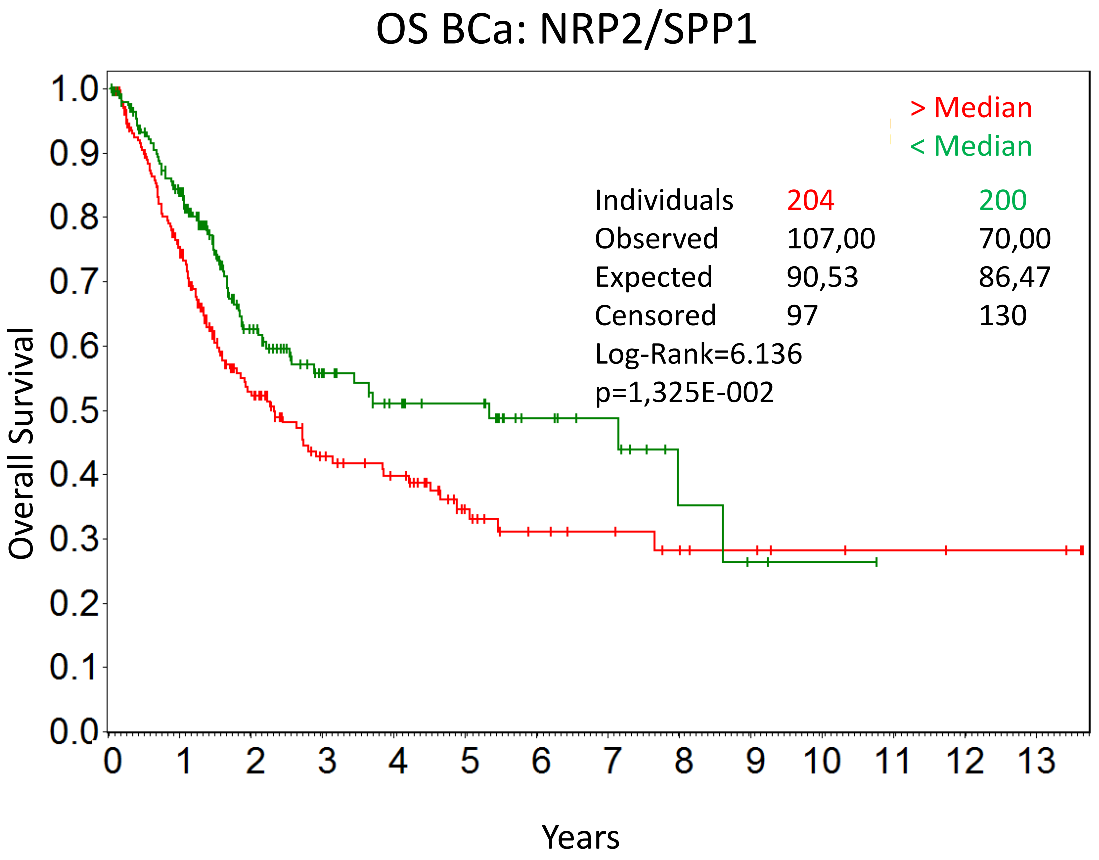

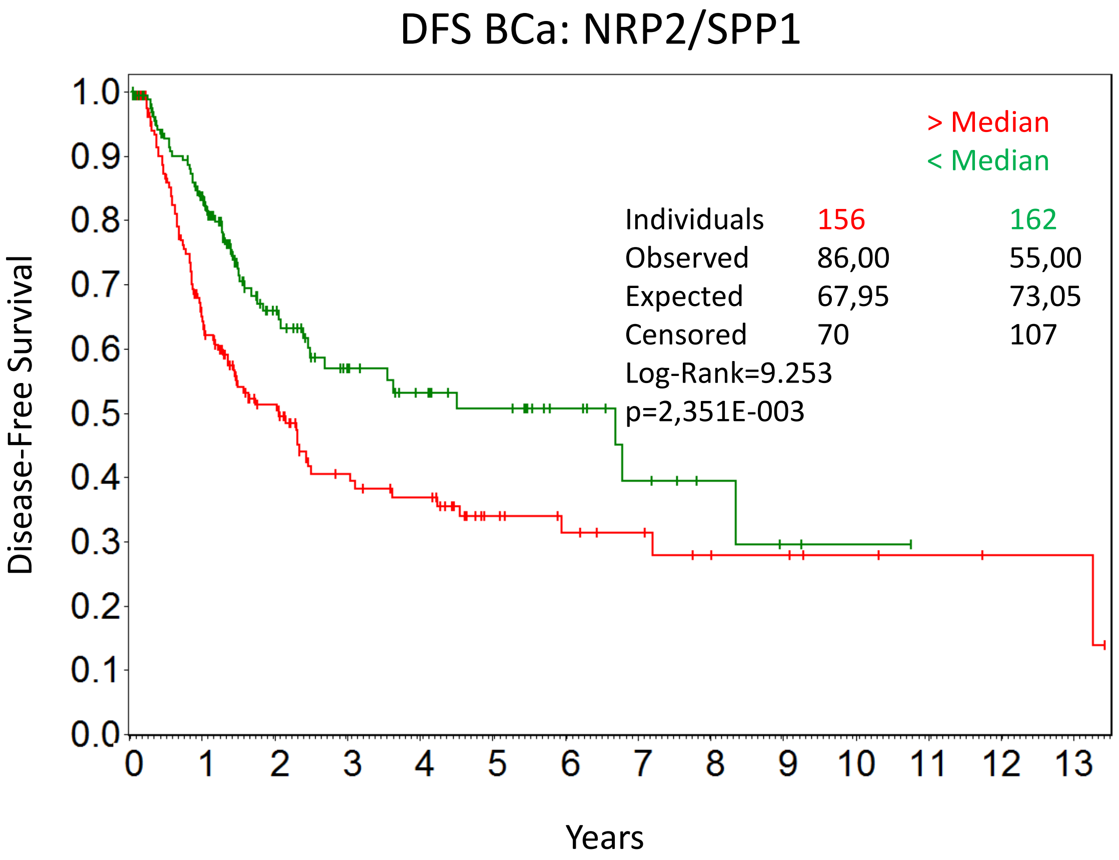

Supplementary Figure 11

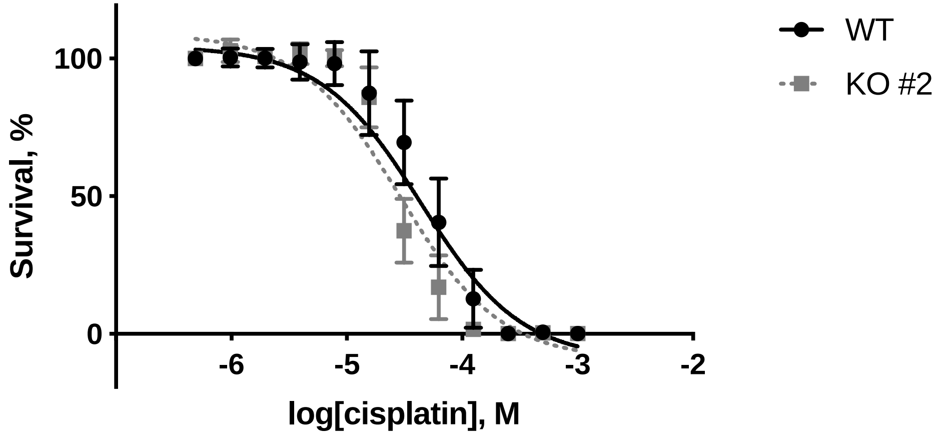

Cells	WT	KO
IC_5, M	$2{,}30 \times 10^{-6}$	$1{,}52 \times 10^{-6}$

Supplementary Figure 12

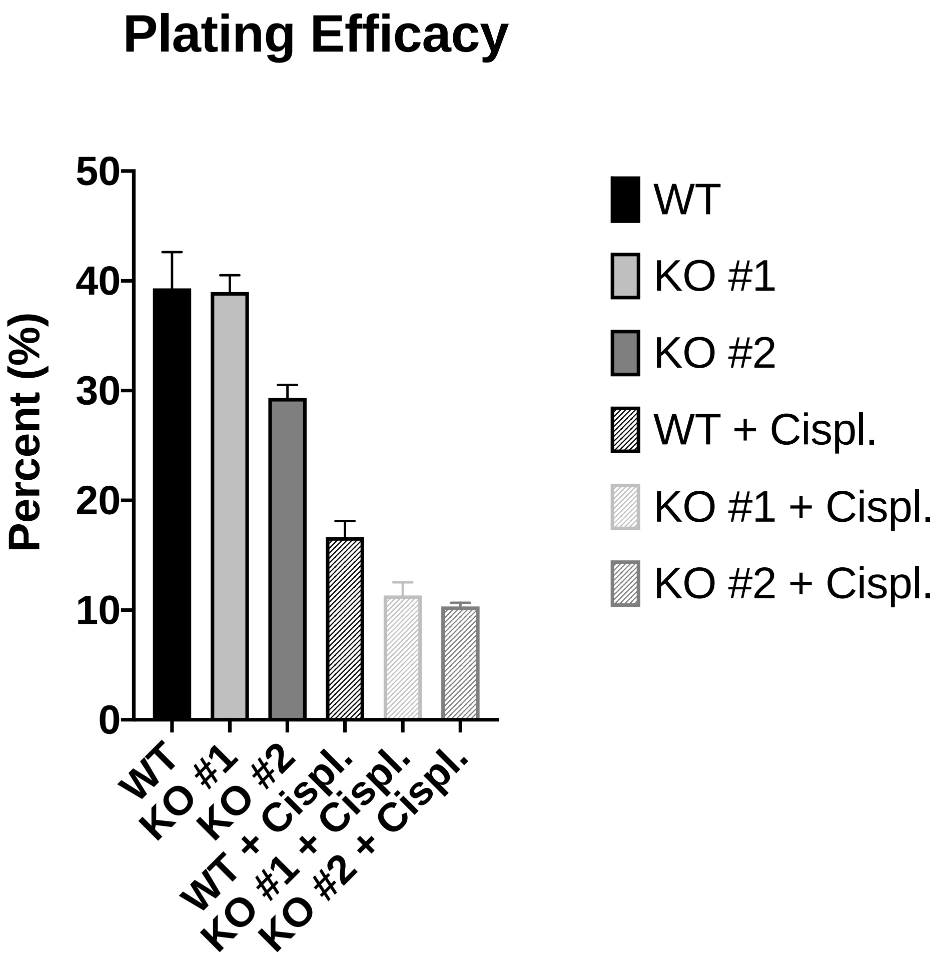

Tukey's multiple comparisons test	Summary	P-value
WT vs. KO #1	ns	>0.9999
WT vs. KO #2	*	0.0154
WT vs. WT + Cispl.	****	<0.0001
WT vs. KO #1 + Cispl.	****	<0.0001
WT vs. KO #2 + Cispl.	****	<0.0001
KO #1 vs. KO #2	*	0.0193
KO #1 vs. WT + Cispl.	****	<0.0001
KO #1 vs. KO #1 + Cispl.	****	<0.0001
KO #1 vs. KO #2 + Cispl.	****	<0.0001
KO #2 vs. WT + Cispl.	**	0.0026
KO #2 vs. KO #1 + Cispl.	***	0.0001
KO #2 vs. KO #2 + Cispl.	****	<0.0001
WT + Cispl. vs. KO #1 + Cispl.	ns	0.32
WT + Cispl. vs. KO #2 + Cispl.	ns	0.1787
KO #1 + Cispl. vs. KO #2 + Cispl.	ns	0.9982

Supplementary Figure 13

Sample	α/b
WT - Cisplatin	4.03
KO #1 - Cisplatin	1.22
KO #2 - Cisplatin	3.65
WT + Cisplatin	21.31
KO #1 + Cisplatin	15.43
KO #2 + Cisplatin	23.69

Supplementary Figure 14

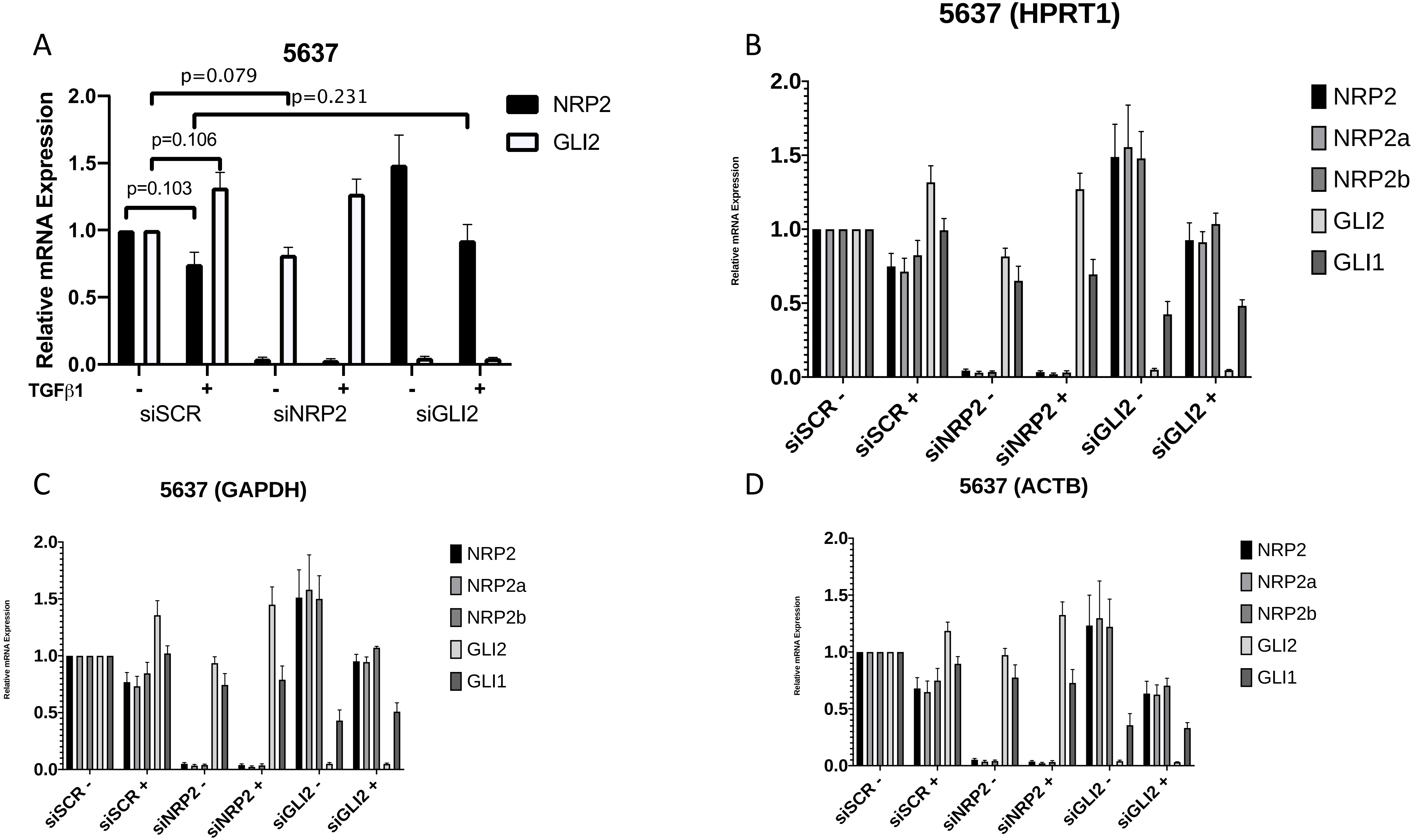

Supplement: Supplementary file 2 [file Presentation_1.pdf]
