# Supplementary material for: Linking NRP2 With EMT and Chemoradioresistance in Bladder Cancer
Source: Front Oncol. 2020 Jan 21;9:1461. doi: 10.3389/fonc.2019.01461 (PMC6986262; doi:10.3389/fonc.2019.01461)
Supplement: Supplementary file 3 [file Presentation_2.pdf]

Supplementary Material

1 Supplementary methods

Cell Proliferation and Cytotoxicity Assay

Cell proliferation and cytotoxicity was analyzed using the Cell Titer Glow luminescent cell viability assay (Sigma-Aldrich, St. Louis, USA). Cells were seeded overnight into 96-well plates at a density of 5×10^4 cells/well and treated with different concentration of cisplatin for 24 h. Cell viability was analyzed by the luminescence levels using a microplate reader (Tecan, Männedorf, Switzerland) according to manufacturer's instructions.

Sphere Forming Assay

Cells were seeded at 2×10^3 cells/well containing 1 ml sphere forming medium in a 24-well ultra-low attachment plate (Corning, New York, USA). Sphere forming medium consisted of mammary epithelial basal medium (Lonza, Basel, Switzerland) supplemented with 1 ml B27 (Invitrogen, Carlsbad, USA), 20 ng/ml EGF (Invitrogen, Carlsbad, USA), 20 ng/ml basic FGF (Peprotech, Rocky Hill, USA), 4 µg/ml Insulin (Invitrogen, Carlsbad, USA) and 1% penicillin/streptomycin (Gibco, life technologies, Waltham, USA). Cells were incubated for 7 days before adding another 1 ml of medium and resuspending spheres using a 1 ml pipette tip. Following another 7 days of incubation, cells were resuspended and images were collected on a Celigo system (Nexcelom Bioscience, Lawrence, USA).

2 Supplementary Figures and Tables

Supplementary Table 1A. Correlation of genes with NRP2 in the provisional BCa cohort of The Cancer Genome Atlas (TCGA)

Supplementary Table 1B. Correlation of genes with GLI2 in the provisional BCa cohort of The Cancer Genome Atlas (TCGA).

Supplementary Table 2. Complete list of p-values for all qPCR experiments as determined by two-way ANOVA.

Supplementary Table 3. Table of PCR primers.

Supplementary Table 4. Table of antibodies.

Supplementary Figure 1. Correlation of GLI2 and NRP2 gene expression in a provisional (A) breast cancer and (B) prostate cancer cohort of The Cancer Genome Atlas (TCGA). (C) mRNA expression levels of NRP2 and Gli2 in a panel of 15 bladder cancer cell lines. Normalized to housekeeping gene HPRT1. (D) Analysis of NRP2 and GLI2 co-expression in the cell lines of urinary tract (n=26) using RNA-sequencing (RNA-seq) data from Broad Institute Cell Line Encyclopedia (https://portals.broadinstitute.org/ccle/page?gene=NRP2; https://portals.broadinstitute.org/ccle/page?gene=GLI2).

Supplementary Figure 2. Kaplan Meier plot of disease-free survival (DFS) of bladder cancer (BCa)

patients with high (red) compared to low mRNA signature (green) for NRP2 (A), GLI2 (B) and combined NRP2/GLI2 (C). Log-Rank value was increased compared to single gene signature.

Supplementary Figure 3. Quantitative real-time PCR of total NRP2, NRP2a, NRP2b, GLI2 and GLI1 in cell line J82: siRNA-mediated knockdown of NRP2 or GLI2 (siNRP2 or siGLI2) or scrambled control (siSCR) and treated with 5 ng/ml TGFβ1 or left untreated (+/-). Normalized to housekeeping gene HPRT1 (A), GAPDH (B) or ACTB (C) and plotted relative to untreated siSCR sample. Significance calculated by two-way ANOVA and shown in supplementary table 2. Error bars indicate standard error of the mean. n=3.

Supplementary Figure 4. Quantitative real-time PCR of total NRP2, NRP2a, NRP2b, GLI2 and GLI1 in cell line HS853T: siRNA-mediated knockdown of NRP2 or GLI2 (siNRP2 or siGLI2) or scrambled control (siSCR) and treated with 5 ng/ml TGFβ1 or left untreated (+/-). Normalized to housekeeping gene HPRT1 (A), GAPDH (B) or ACTB (C) and plotted relative to untreated siSCR sample. Significance calculated by two-way ANOVA and shown in supplementary table 2. Error bars indicate standard error of the mean. n=3.

Supplementary Figure 5. (A) Western blot analysis of NRP2 expression in parental wildtype bladder cancer cell line RT112 and KO clone #2 which were treated with 5 ng/ml TGFβ1 or left untreated (+/-). (B) Vimentin expression induced by TGFβ1. Data of the PCR array. n=2.

Supplementary Figure 6. (A) SPP1 expression in human EMT PCR array relative to untreated WT samples. n=2. Error bars indicate fold change uncertainty = σFC = FC * ln2 * sqrt(σx2 / nx + σy2 / ny). Correlation plot for all detected genes on the panel comparing KO #1 to WT (B) or KO #2 to WT (C). Red arrow points to SPP1 which was consistently deregulated in both independent knockout clones. n=2. Normalized to housekeeping gene HPRT1.

Supplementary Figure 7. Quantitative real-time PCR of CDH1 (E-Cadherin) normalized to housekeeping gene HPRT1 and plotted relative to treated WT sample. Significance calculated by two-way ANOVA. No significance detected. Error bars indicate standard error of the mean. n=4.

Supplementary Figure 8. (A) Sphere Forming Assay in defined anchorage-independent conditions. Significance determined by two-tailed, unpaired student's T-test. Error bars indicate standard error of the mean. n=3. (B) qPCR of EMT regulators following NRP2 knockdown in cell line HS853T without TGFβ1 treatment. Gene expression was normalized to housekeeping genes HPRT1 and GAPDH. Significance calculated by paired t-test. Error bars indicate standard error of the mean. n=3. (C) Validated genes positively correlate with expression of both, NRP2 and GLI2 genes in a provisional bladder cancer cohort of The Cancer Genome Atlas (TCGA), n = 408. (D) Surviving fraction of cell line J82 after transfection with scrambled siRNA or SPP1 specific siRNA. Error bars indicate standard deviation. n=3. (E) SPP1 knockdown was validated by qPCR. Significance calculated by paired t-test. Error bars indicate standard error of the mean. n=3.

Supplementary Figure 9. Kaplan Meier plot of overall survival (OS, left) and disease-free survival (DFS, right) of bladder cancer (BCa) patients with high (red) compared to low mRNA signature (green) for SPP1 (Osteopontin).

Supplementary Figure 10. Kaplan Meier plot of overall survival (OS, left) and disease-free survival (DFS, right) of bladder cancer (BCa) patients with high (red) compared to low mRNA signature (green) for combined NRP2/SPP1 signature improved predictive value for disease-free but not overall survival compared to single NRP2 gene expression.

Supplementary Figure 11. Cell viability analysis after treatment with different doses of cisplatin. From this curve, IC5 doses were calculated and lower dose (corresponding to KO #2) was applied in colony formation assay. n=3.

Supplementary Figure 12. Plating efficacy (left) with corresponding values for significance (right). Significance determined by ordinary one-way ANOVA. Error bars indicate standard error of the mean. n=3.

Supplementary Figure 13. Summary of alpha-beta ratio of parental wildtype bladder cancer cell line RT112 , NRP2 KO clone #1 and NRP2 KO clone #2 treated with cisplatin at concentrations of 1.52×10-6 M for 24 h or untreated. The alpha-beta ratio are defined from interpolation of linear-quadratic cell survival curves using mean values of three independent experiments.

Supplementary Figure 14. (A) Quantitative real-time PCR of NRP2 and GLI2 in cell line 5637 normalized to HPRT1. (B, C, D): qPCR of total NRP2, NRP2a, NRP2b, GLI2 and GLI1 in cell line 5637: siRNA-mediated knockdown of NRP2 or GLI2 (siNRP2 or siGLI2) or scrambled control (siSCR) and treated with 5 ng/ml TGFβ1 or left untreated (+/-). Normalized to housekeeping gene HPRT1 (B), GAPDH (C) or ACTB (D) and plotted relative to untreated siSCR sample. Significance calculated by two-way ANOVA and shown in supplementary table 2. Error bars indicate standard error of the mean. n=3.
